# Fenton‐Inactive Cd Enables Highly Selective O_2_‐Derived Domino Reaction

**DOI:** 10.1002/advs.202407051

**Published:** 2024-11-08

**Authors:** Yitong Wang, Huilin Wang, Yulu Yang, Zhaomin Hao, Ruiping Deng, Qingsong Dong, Qingchao Liu, Hongpeng You, Shuyan Song

**Affiliations:** ^1^ School of Chemistry and Chemical Engineering Nanchang University Nanchang 330031 P. R. China; ^2^ Ganjiang Innovation Academy Chinese Academy of Sciences Ganzhou 341000 P. R. China; ^3^ Changchun Institute of Applied Chemistry Chinese Academy of Sciences Changchun 130022 P. R. China; ^4^ College of Chemistry Zhengzhou University Zhengzhou 450001 P. R. China

**Keywords:** electroreduction, fenton‐inactive Cd, selective oxidation

## Abstract

Advancing and deploying Fenton‐inactive Cd that combines excellent catalytic activity, selectivity, and stability remains a serious challenge, predominantly owing to the difficulty in regulating the intrinsic electronic states and local geometric structures of such fully occupied *d* ^10^ *s* ^2^ configuration. In this work, a combination of experiments and theoretical calculations reveals that the incorporation of boron (B) enables the tuning of the average oxidation state of Cd^0^ to Cd^δ+^, facilitating electron localization and implementing a different electrocatalytic preference compared to conventional *d* ^10^‐electron configurations. The resulting Cd(B) catalyst demonstrates high selectivity (>90% on average) in the O_2_‐to‐H_2_O_2_ conversion, negligible activity loss over 100 h, and a superior H_2_O_2_ production rate (15.5 mol∙g_cat_^−1^ h^−1^ at −100 mA). More unexpectedly, the in situ generated H_2_O_2_ exhibits a unique advantage over commercial products, selectively oxidizing cinnamaldehyde to benzaldehyde by modulating the practical current.

## Graphical abstract

In this work, the preparation of a *d* ^10^‐electron catalyst is reported by combining Cd and B. The resulting Cd(B) catalysts demonstrate notable selectivity toward the 2*e* ^−^ ORR and exhibit improved catalytic activity for H_2_O_2_ production, along with exceptional stability. More unexpectedly, the in situ generated H_2_O_2_ displays a distinct advantage over commercial products as it exhibits selective oxidation of cinnamaldehyde to benzaldehyde through modulation of the practical current.

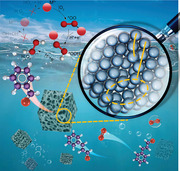

## 1 Introduction

**Figure 1:**
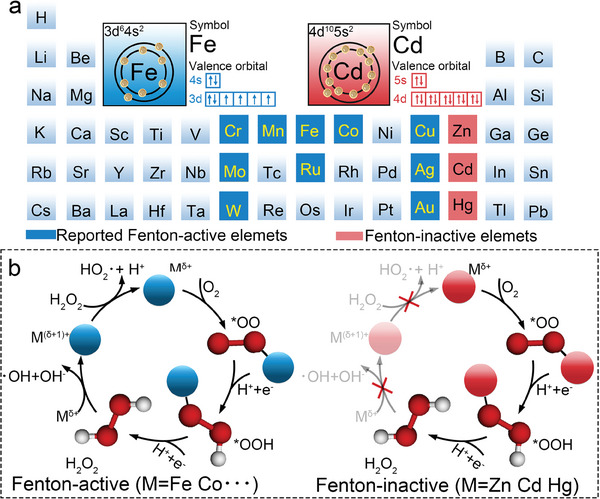
Illustration of Fenton‐active and Fenton‐inactive catalysts. a) Widely reported Fenton‐active (represented by Fe) and Fenton‐inactive (represented by Cd) transition‐metal element. b) Comparison of mechanism for H_2_O_2_ formation and degradation by Fenton‐active and Fenton‐inactive catalysts.

Development of cost‐effective and high‐performance transition‐metal catalysts for highly selective oxygen (O_2_)‐derived reaction, especially at domino approaches from O_2_ to hydrogen peroxide (H_2_O_2_) and then to organic products,^[^ ^1^ ^]^ in which the intermediate does not require separation and purification, achieving atomic economy and environmental friendliness.^[^ ^2^, ^3^, ^4^, ^5^ ^]^ Expertise from the catalysis landscape, the aforementioned topic remained significantly challenging, largely due to the dilemma of mitigating the paradoxical properties of current Fenton‐active and Fenton‐inactive catalysts. Conventional Fenton‐active catalysts, in which the sources are mainly from Fe, Co and Cu, etc., have proved to be active for two‐electron oxygen reduction reaction (2*e* ^−^ ORR) due to their advantages of partially occupied *d* orbitals and suitable *d*‐band configuration.^[^ ^6^, ^7^, ^8^, ^9^ ^]^ However, given advances in Fenton‐active catalysts, an outstanding challenge is that these metal‐active sites can provide an attractive O_2_‐to‐H_2_O_2_ approach while still degrading H_2_O_2_ to ^*^OH (**Figure** **1**).^[^ ^10^ ^]^ Moreover, the intrinsic properties of partially occupied *d* orbitals frequently lead to sacrificing chemical stability, which greatly limits the large‐scale application of Fenton‐active catalysts for 2*e* ^−^ ORR.^[^ ^11^, ^12^, ^13^ ^]^ Accordingly, there is a growing interest in advancing and deploying Fenton‐inactive catalysts to get rid of the dilemma of Fenton‐active catalysts.

As expected, most previously reported matrices that are composed of *M*, *M*–O_x_, *M*–N_4,_ and *M*–N–C (*M*, zinc group element; C/N, carbon/nitrogen‐supported atoms), etc., have natural advantages in avoiding the dilemma of production‐degradation synchronism in Fenton‐active catalysts, as well as retaining durability in harsh environments owing to the superior *d* ^10^‐electron configuration.^[^ ^10^, ^12^, ^14^ ^]^ However, these Fenton‐inactive catalysts are simultaneously outweighed by the drawbacks of inferior catalytic activity, mainly because such a fulfilled *d* ^10^‐electron configuration and the resultant *d*‐band structure would severely limit the movement of electrons. The promising candidacy of main group metals as Fenton‐inactive catalysts are also recognized. Nevertheless, the use of main group metals as the primary active centers of catalysts often leads to stable and relatively inert electronic structures, which frequently limits overall performance enhancement.^[^ ^14^, ^15^ ^]^ Recent advancements in certain Zn‐based catalysts could offer theoretical models for the enhancement of catalytic activity by tuning *s*‐electrons and local geometric structures.^[^ ^16^, ^17^ ^]^ However, such Zn‐based Fenton‐inactive catalysts have not yet been realized to demonstrate encouraging selectivity toward 2*e* ^−^ ORR, making the development of more efficient electrocatalysts an important but also challenging problem.

By comparison to the Zn element, Cd holds a similar *d* ^10^‐electron configuration while it has one more electronic layer, which enables a more sensitive property to the variation of the local environment.^[^ ^18^ ^]^ Meanwhile, boron (B, [He]2*s* ^2^2*p* ^1^) — a unique metalloid in which the single 2*p* electron would favor metallicity but its orbital radius is close to that of the 2*s* state — in past reports, has been favored to influence the binding affinity of the surface and metal activity in light of such unique electronic structure.^[^ ^19^ ^]^ Herein, we report on the preparation of a *d* ^10^‐electron catalyst by combining Cd and B, in which incorporating B into Fenton‐inactive Cd leads to the disruption of periodicity in the local lattice, and defects were successfully captured based on atomic‐level technology. Arising from the incorporated B, the evidence from X‐ray absorption near‐edge structures (XANES) of Cd K‐edge suggested that the original environment of neighboring atoms has been altered and the incorporated B could offer the ability to tune the average oxidation state of Cd^0^ to Cd^δ+^. The resultant Cd(B) catalysts exhibit high selectivity toward the 2*e* ^−^ ORR and show enhanced catalytic activity (15.5 mol g_cat_^−1^ h^−1^ at −100 mA) for H_2_O_2_ production and excellent stability (≈100 h). Moreover, the in situ generated H_2_O_2_ subsequently exhibits a unique advantage over commercial H_2_O_2_ in selectively oxidizing *α*, *β*‐unsaturated aldehydes, presenting a valuable and distinctive application.

## 2 Results and Discussion

### 2.1 Preparation and Characterization of Cd(B)

**Figure 2:**
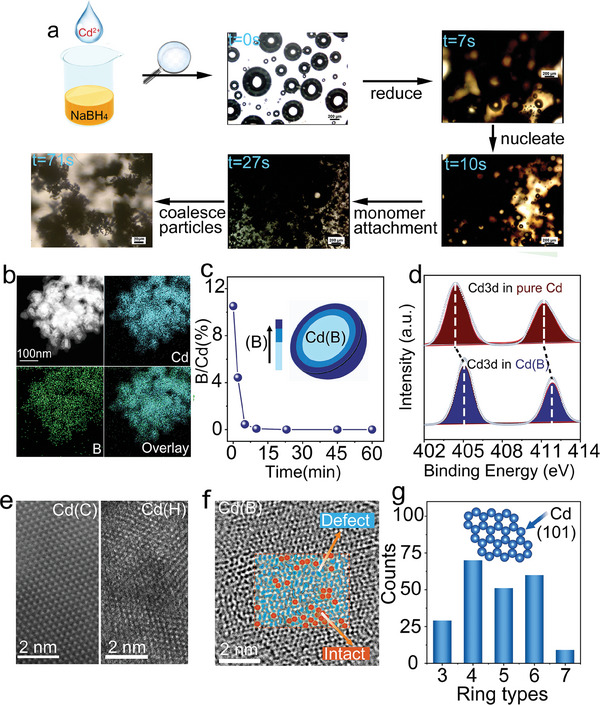
Preparation and characterization of Cd(B) powders. a) Monitoring the in situ reduction of Cd^2+^ by NaBH_4_ under optical microscopy. b) Elemental mapping. c) Dissolving‐time‐dependent B concentrations of the Cd(B) sample as measured by ICP‐OES, indicating that B is present on the surface of Cd. d) Comparison of Cd3*d* XPS patterns between Cd(B) and pure Cd e) ADF‐TEM image of Cd(C) and Cd(H); f) ADF‐TEM image of Cd(B). g) Statistics of different types of Cd‐stacking rings calculated from randomly selected ADF‐TEM images of Cd(B).

Cd(B) powders were prepared using a one‐pot wet‐chemical approach, in which NaBH_4_ was used to reduce Cd^2+^ ions. The resultant Cd(B) sample has a porous dendritic morphology with nanostructured features on the scale of 80–150 nm (Figure S1, Supporting Information). To verify the formation mechanism of Cd(B), an advanced research‐grade optical microscope was utilized to visualize the reduction process of Cd^2+^ by NaBH_4_ and observe the subsequent formation of Cd(B). Generally, the formation of Cd(B) involves nucleation and crystal growth, wherein the abundance of bubbles generated during the vigorous reaction leads to the formation of pore structures (**Figure** **2**a). Power X‐ray diffraction (XRD) suggested that the peaks could be exclusively indexed to the hexagonal crystal structure of Cd, which corresponds to JSPCD no. 05–0674 (Figures S2 and S3, Supporting Information). To gain insight into the effect of reductants, two synthetic protocols with trisodium citrate and hydrazine hydrate were chosen for comparison, one resulting in Cd(C) with a block‐like structure and another Cd(H) with a microsphere of particles, as revealed by scanning electron microscopy (SEM) (Figures S4–S7, Supporting Information). For the sample of Cd(B), the elemental mapping images demonstrate the coexistence and uniform distribution of Cd and B elements (Figure 2b). To probe the distribution of B in Cd(B) samples, we employed time‐dependent inductively coupled plasma optical emission spectroscopy (ICP‐OES) to explore the function of depth within the Cd‐based catalyst, which revealed that the B could be gradually etched away with the increase of acid corrosion, suggesting that the incorporated B is mainly distributed on the surface of Cd(B) (Figure 2c; Figure S8, Supporting Information). This phenomenon is governed by the kinetics and thermodynamics of the reduction process and is attributed to the significant energy difference between surface and internal atoms within the Cd material.^[^ ^20^ ^]^ Surface atoms generally possess higher energy due to their lower coordination with neighboring atoms.^[^ ^21^ ^]^ Consequently, B atoms or ions preferentially bond with reduced Cd atoms on the surface, leading to surface doping. In addition, the rate of surface doping is likely much faster than the diffusion rate of B atoms or ions into the bulk phase of Cd, a process further limited by the inherently low diffusivity of boron.^[^ ^21^ ^]^ Moreover, X‐ray photoelectron spectroscopy (XPS) was used to detect the surface composition of the as‐synthesized Cd(B) sample (Figure S9, Supporting Information). Similar to the conclusion from the aforementioned measurements, the XPS spectrum confirmed that B‐incorporated Cd was successfully prepared using our synthesis protocol. In addition to providing experimental evidence for B identification in Cd(B), XPS measurements were conducted to further examine the differences in surface compositions and electronic states for Cd3*d*. A significant upshift of Cd3*d* peaks for Cd(B) was observed when it was compared with pure Cd (Figure 2d), suggesting that the surfaces of two Cd samples are different in electronic structure.

To illustrate a more elaborate and accurate structure of Cd(B), a spherical aberration‐corrected transmission electron microscope (AC‐TEM) was employed to study the surface and local geometric structures. Figure 2e shows annular dark‐field transmission electron microscopy (ADF‐TEM) images of the counterparts of Cd(C), in which no obvious defect or lattice deformation is observable, manifesting the high crystallinity of these samples. Localized defects or lattice deformations are observed in the Cd(H) catalysts, which may be attributed to the prolonged reduction of cadmium salts. This extended process likely promotes regular lattice growth through interactions with hydrazine hydrate.^[^ ^22^ ^]^ In stark contrast, copious amounts of defects and topological disorders were successfully captured for Cd(B), which presented a typical class of irregular structure due to the introduction of heteroatoms, which presented a typical class of irregular structure due to the introduction of heteroatoms. (Figure 2f). To quantitatively describe the defective structures, we mapped different types of Cd‐stacking rings by colors, in which the hexagon from a perfect (101) facet are highlighted in blue while other shapes are green, respectively. Figure 2g shows the statistics of different types of Cd‐stacking rings calculated from randomly selected ADF‐TEM images, indicating that the proportion of defects is relatively large. Moreover, the well‐defined diffraction rings in the selected area electron diffraction (SAED) pattern presented the polycrystalline nature of Cd(B) and proved the changes of facets in comparison with the lattice distance in standard Cd (Figure S10, Supporting Information), which could be found for a similar phenomenon in other incorporated materials.^[^ ^23^, ^24^ ^]^

**Figure 3:**
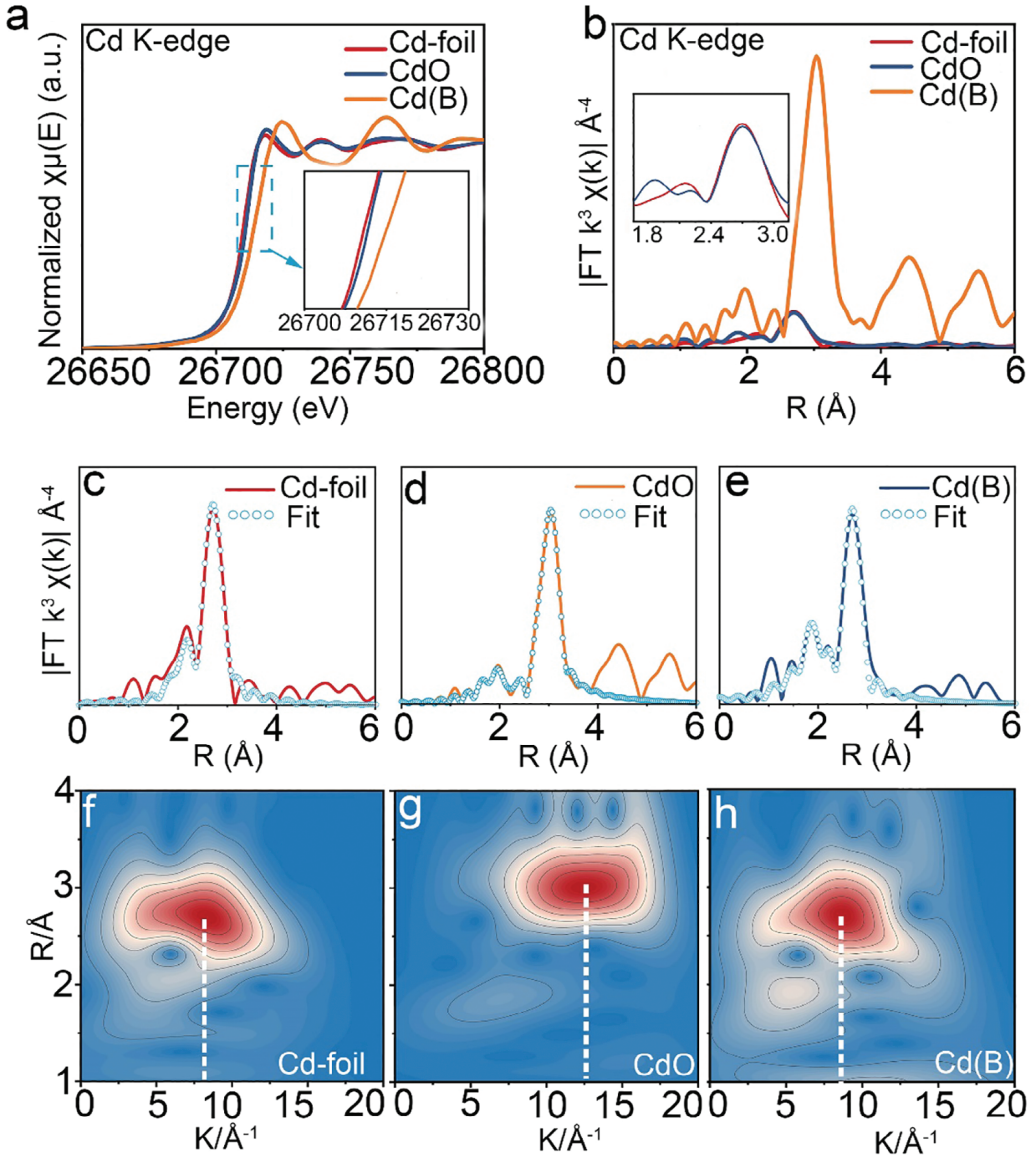
Structural analysis of Cd(B), referenced Cd‐foil and CdO. a) Normalized Cd K‐edge XANES spectrogram. b) FT‐EXAFS spectrum. c–e) EXAFS fitting curves at the R space. f–h) WT of Cd K‐edge EXAFS spectra.

To directly probe the chemical state and local environment, Cd(B) was subsequently characterized by X‐ray absorption near‐edge spectroscopy (XANES) and extended X‐ray absorption fine structure (EXAFS) measurements. The XANES spectrum is frequently used to determine the position of the absorption edge, which can be defined as the maximum of the first derivative to represent the oxidation state of Cd. As shown from the normalized Cd K‐edge XANES spectrogram (**Figure** **3**a), the position of the absorption edge in Cd(B) is between Cd‐foil and CdO, indicating that the average oxidation state of Cd in Cd(B) is between the metal Cd^0^ and Cd^2+^. To identify specific data, the oxidation state of Cd(B) was taken as a function of the energy displacement for the Cd K edge (Figure S11, Supporting Information), and it can be concluded that the average oxidation state is +0.496, which means that the Cd atom in Cd(B) has unpaired electrons in the 5*s* orbital. In the Fourier‐transformed EXAFS (FT‐EXAFS) spectrum and its fitting curve, three peaks were detected near 1.86, 2.22, and 2.70 Å, respectively (Figure 3b–e; Figures S12 and S13, Supporting Information). The peak near 1.96 Å in the FT‐EXAFS spectrum of CdO could correspond to the Cd‐O coordination, while the peak near 1.86 Å in Cd(B) could be attributed to the formation of the Cd─B bond.

Achieving deep insight into electronic states and local geometric structures requires a detailed understanding of the coordination environment in Cd(B). In this study, wavelet transform (WT) was conducted to analyze the EXAFS oscillations of the Cd K edge. The maximum intensity of the WT contour plot for Cd‐foil and CdO presented ≈8.05 and 12.45 Å^−1^. In contrast, as shown in the WT contour plot of Cd(B), its maximum intensity (≈8.60 Å^−1^) was located between Cd‐foil and CdO, which could be attributed to the coordination of Cd and B (Figure 3f–h). As previously reported, it is easier for Cd to form a pentacene structure with the surrounding atoms because the atomic radius of the Cd atom (1.52 Å) is larger than that of well‐documented transition‐metal atoms, such as Fe (1.26 Å), Co (1.25 Å) and Ni (1.25 Å), etc.^[^ ^25^ ^]^ According to the fitting results of the ARTEMIS program in IFEFFIT, the coordination number of Cd‐B was 4.79, which was similar to the reported literature (Table S1, Supporting Information).^[^ ^25^ ^]^ Nonetheless, 4.79 is less than 5 and this result indicated that the coordination of Cd‐B in Cd(B) was unsaturated, which could provide open sites for the adsorption of O_2_ molecules to enable catalyst refinement toward ORR.

### 2.2 O_2_‐Derived Domino Reaction

**Figure 4:**
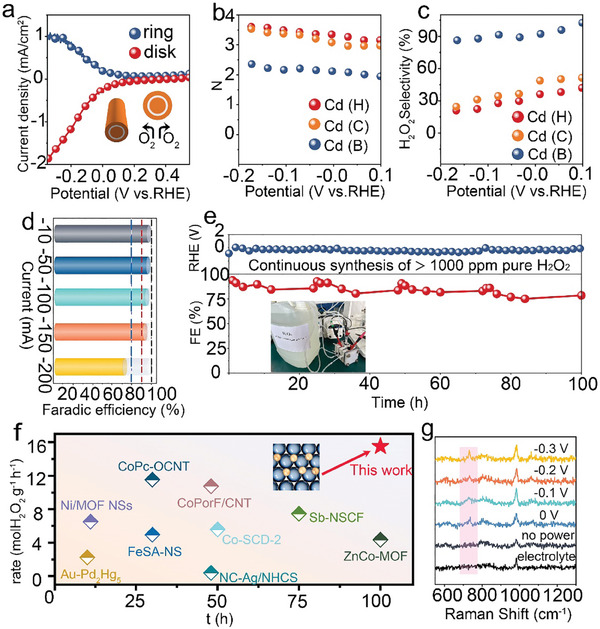
Evaluation of the electrochemical ORR performance of Cd(B). a) Linear sweep voltammetry of RRDE measurement at 900 rpm in O_2_‐saturated electrolyte at 5 mV s^−1^ with the ring and disk current density. b) The corresponding average number of transferred electrons (N). c) Calculated H_2_O_2_ selectivity (%). d) The concentrations and faradaic efficiencies of H_2_O_2_ at different operating currents for 1 h by a solid‐electrolyte cell. e) Durability performance of Cd(B) at −100 mA in the O_2_‐saturated electrolyte. f) Comparison of the 2*e* ^−^ ORR performance for Cd(B) with similar materials. g) In situ Raman signals of Cd(B) at different biases.

The derived unique structures and resulting electronic states of Cd(B) provide new opportunities to explore the activity of ORR. To test the practical performance of Cd(B), the ORR performance was first examined in O_2_‐saturated 0.1 m K_2_SO_4_ using a rotating ring‐disk electrode (RRDE). The O_2_ reduction and H_2_O_2_ oxidation current were measured on the disk and the platinum ring electrode respectively. To quantify the amount of H_2_O_2_, the potential of the ring electrode was fixed at 1.2 V (vs the reversible hydrogen electrode, RHE) to avoid other ORR currents at the ring electrode, allowing only H_2_O_2_ oxidation. As evidenced by the polarization curves (**Figure** **4**a; Figures S14 and S15, Supporting Information), Cd(B) exhibits significantly higher ring current and lower overpotential compared to its counterparts, including Cd(C), Cd(H), Cd(B)‐1, Cd(B)‐2, and Cd(B)‐3, indicating excellent catalytic activity for oxygen reduction reaction (ORR). Furthermore, to obtain a better understanding of the electrocatalytic activity for 2*e* ^−^ ORR, we estimated the electrochemical surface area (ECSA). Remarkably, Cd(B) with the smallest ECSA demonstrates relatively superior 2*e* ^−^ ORR performance, suggesting a potential correlation with the inherent characteristics of the catalyst that are not directly linked to ECSA (Figure S16, Supporting Information). Known electrocatalytic O_2_ reduction can follow either a 2*e* ^−^ pathway to produce H_2_O_2_ (Equation 1) or a competing 4*e* ^−^ transfer process (Equation 2), thereby reducing oxygen to H_2_O. To clarify the affiliation of Cd(B), the corresponding electron transfer numbers and selectivity were plotted as a function of potential (Figure 4b,c).

(1) $$O_{2}+2H^{+}+2e^{-}\to H_{2}O_{2}\;$$

(2) $$O_{2}+4H^{+}+4e^{-}\to H_{2}O\;$$

Herein, the results indicate that the Cd(B) could offer a 2*e* ^−^ ORR pathway and high H_2_O_2_ selectivity (>90% on average), while another two Cd catalysts of synthetic protocols with trisodium citrate and hydrazine hydrate mainly facilitate the 4*e* ^−^ ORR pathway for H_2_O production.

To quantify the catalytic activity of Cd(B), comparisons of the H_2_O_2_ concentrations and corresponding faradaic efficiency (FE) that were produced at different operating currents by a solid‐electrolyte cell were presented (Figure 4d; Figures S17–S20, Supporting Information). Overall, the Cd(B) catalyst could show over 90% FE for H_2_O_2_ production in a wide range of currents (−10 mA, 98.96%; −50 mA, 97.5%; −100 mA, 95.93%; −150 mA, 91%). When the operating current increased to −200 mA, the FE was significantly lower than 80%, predominantly owing to the limitation of commercial gas diffusion electrodes being prone to flooding under high currents. To assess the long‐term stability of Cd(B) during 2*e* ^−^ ORR, the galvanostatic measurements are conducted on the electrodes at −100 mA in the O_2_‐saturated electrolyte. Presenting the performance of continuous synthesis of > 1000 ppm H_2_O_2_, the curves of FE and bias, along with the comparison of XPS and XRD before and after 2*e* ^−^ ORR, indicated that the Cd(B) could maintain good catalytic activity for at least 100 h, which not only demonstrates high activity but also retains the unique advantage of most of the *d* ^10^‐electron catalysts in terms of stability (Figure 4e; Figures S21 and S22, Supporting Information). The electrocatalytic reduction of O_2_ in an aqueous solution exhibited comparable or even superior performance to some reported metal catalysts, such as Au‐Pd_2_Hg_5_ (1.49 mol g_cat_^−1^ h^−1^),^[^ ^26^ ^]^ Sb‐NSCF (7.46 mol g_cat_^−1^ h^−1^),^[^ ^27^ ^]^ Co‐SCD‐2 (5.58 mol g_cat_^−1^ h^−1^),^[^ ^24^ ^]^ etc., which have demonstrated the effectiveness of Fenton‐inactive Cd as a highly efficient catalyst for electrochemical synthesis of H_2_O_2_ from O_2_ (Figure 4f; Tables S2 and S3, Supporting Information).

Exploring the direct evidence of ORR and the nature of the electrocatalytic activity is ultimately necessary for providing a stable and affordable approach in light of the switch between current catalysts and future efforts. To probe the type of binding sites, in situ electrochemical surface‐enhanced Raman spectroscopy (SERS) was used to dynamically detect energetic fingerprints of the binding sites on the Cd(B). In this study, we obtained preliminary results that in situ 2*e* ^−^ ORR could lead to a notable difference in SERS ≈731 cm^−1^ (no power/pure electrolyte as references) when the bias was applied in the −0.3–0 V (vs RHE) range, which could be attributed to the formation of the O‐contained metal intermediates (Figure 4g).^[^ ^28^, ^29^ ^]^ With regard to 2*e* ^−^ ORR, H_2_O_2_ could be generated by either a direct two‐electron reduction or a sequential two‐step single‐electron O_2_ reduction. To provide evidence for elucidating the pathway of electrochemical O_2_‐to‐H_2_O_2_ synthesis, quenching experiments were carried out in the presence of a scavenger (1,4‐benzoquinone, *p*‐BQ) for ^*^OO following reported works.^[^ ^30^ ^]^ In this work, the H_2_O_2_ production could be completely quenched in the presence of 2.0 mm *p*‐BQ (Figure S23, Supporting Information). These findings indicated that the electrochemical O_2_‐to‐H_2_O_2_ synthesis with Cd(B) catalysts follows the two‐step single‐electron pathway, in which superoxide radicals could be involved as important intermediates of 2*e* ^−^ ORR.

**Figure 5:**
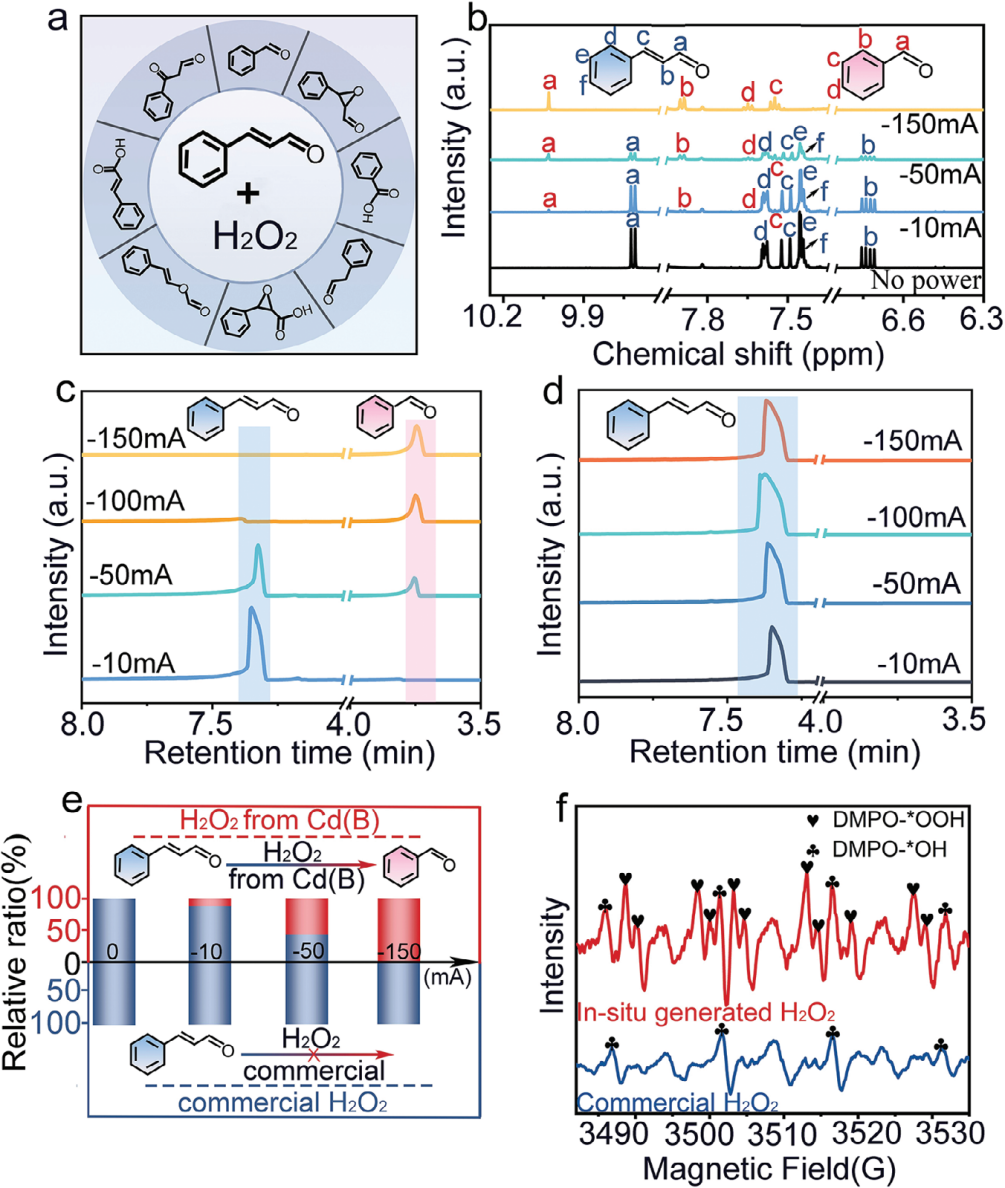
Oxidation reaction between H_2_O_2_ and cinnamaldehyde. a) Reported organic products from the oxidation reaction of H_2_O_2_ and cinnamaldehyde. b,c) ^1^HNMR spectrum and GC‐MS under different operating currents with in situ generated H_2_O_2_ as oxidant. d) GC‐MS under the same concentration of commercial H_2_O_2_ as oxidant. e) Comparison of the result of selective oxidation, and the vertical axis is the concentration ratio of benzaldehyde (red) to cinnamaldehyde (blue). f) EPR curve of DMPO‐^*^OOH and DMPO‐^*^OH formed in the aqueous solution of in situ generated H_2_O_2_.

For practical applications of the product, the in situ generated H_2_O_2_ was subsequently used for the oxidation of *α*, *β*‐unsaturated aldehydes,^[^ ^31^ ^]^ in which both double bonds and aldehyde groups can serve as oxidized sites. In this work, as exemplified by cinnamaldehyde, previous reports suggest that various types of organic products would be produced after the oxidation reaction with H_2_O_2_ (**Figure** **5**a).^[^ ^32^ ^]^ Under the same concentration of cinnamaldehyde, we investigated the nuclear magnetic resonance hydrogen spectrum (^1^HNMR) and gas‐chromatography‐mass spectrometry (GC‐MS) in the products under different operating currents (Figure 5b,c). Unexpectedly, the ^1^HNMR spectrum and GC‐MS indicated that cinnamaldehyde can be converted into the only organic product — benzaldehyde, demonstrating that in situ generated H_2_O_2_ enables highly selective oxidation reaction. In parallel, when the same concentration of commercial H_2_O_2_ is compared under similar conditions, we find that GC‐MS results show that commercial H_2_O_2_ does not undergo the oxidation process and no new organic molecules are produced (Figure 5d,e). Noting the differentiation of in situ generated H_2_O_2_ in this work, 5,5‐dimethyl‐1‐pyrroline N‐oxide (DMPO) — an efficient free radical scavenger with a stable ability of electronic capture, was used to study the intermediates of DMPO‐^*^OOH and DMPO‐^*^OH. In the experiment, both ^*^OOH and ^*^OH radicals were effectively captured by DMPO within the Cd(B)‐driven electrosynthesis system (Figure 5f). The intensity of the DMPO‐^*^OOH peak was found to significantly exceed that of the DMPO‐^*^OH peak, suggesting that the primary intermediate produced by the Cd(B) electrocatalyst is ^*^OOH. In contrast, only a weak DMPO‐^*^OH signal was detected in the commercially available H_2_O_2_. Consequently, we postulated that the selective oxidation of cinnamaldehyde to benzaldehyde probably could be attributed to the presence of a large amount of ^*^OOH in the in situ generated H_2_O_2_, which has been reported with a similar phenomenon in other materials.^[^ ^32^ ^]^

### 2.3 Density Functional Theory (DFT) Calculations

**Figure 6:**
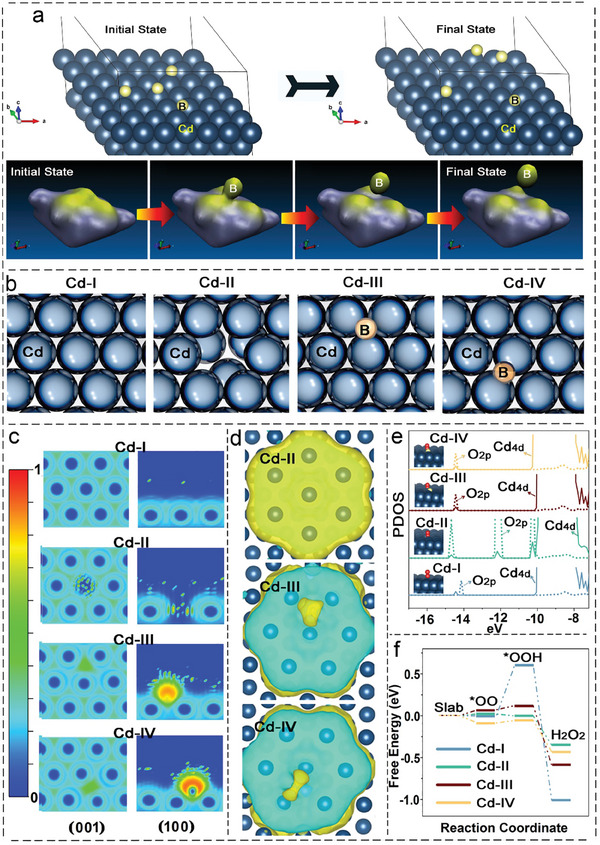
DFT simulations. a) The results of molecular dynamics. b) Atomic models for DFT calculation. c) Comparison of ELF results. d) Images of charge density difference. Yellow and cyan isosurfaces show electron gain and electron loss, respectively. e) Calculated PDOS. f) Diagram of calculated free energy.

Theoretical calculations are of great significance to the selection of catalysts and the understanding of reaction mechanisms.^[^ ^33^ ^]^ To explore theoretical insight into local geometric structures and electrocatalytic activities, DFT studies were performed using the Vienna Ab initio Simulation Package (VASP). Combining the results of molecular dynamics, four atomic models (denoted as Cd‐I, Cd‐II, Cd‐III, and Cd‐IV) were listed to compare the differences in the surface structure between intact and defective Cd (**Figure** **6**a,b). Electron localization function (ELF) is one of the important ways to study electronic structures, which can effectively characterize the degree of electron localization in catalysts. The intact Cd (Cd‐I) exhibit strong electronic localization, while different electron delocalization properties could be detected at defect sites where Cd atoms are lost (Cd‐II) and heteroatom of B is introduced (Cd‐III and Cd‐IV) (Figure 6c), which suggested that the defective models (Cd‐II, Cd‐III, and Cd‐IV) regulate the electronic structure of intact Cd active sites. From the image of charge density difference, convincing new evidence supports the fact that different Cd‐based catalysts could be designed by the doping of B atoms. As shown in Figure 6d, electron accumulation mainly occurs on the surface where Cd atoms are missing in Cd‐II, and similar electron transfer is more centralized on the non‐metal atoms of B in Cd‐III and Cd‐IV. This charge transfer and rearrangement in Cd‐II, Cd‐III, and Cd‐IV implies that *d* ^10^‐electron Cd could be explored as potential catalysts with superior catalytic activity.

To gain molecular‐level insight into the effect of intermediates binding, we further calculated the projected density of states (PDOS) of Cd4*d* and O2*p* orbitals based on the above four atomic models. In addition to presenting distinct PDOS plots from intact Cd (Cd‐I), our findings demonstrate a high overlap between the binding states of O2*p* and Cd4*d* when ^*^OO is absorbed onto the surface of defective Cd‐II, Cd‐III, and Cd‐IV (Figure 6e). Besides, the *d*‐band center of the Cd‐II (−8.604 eV), Cd‐III (−8.616 eV), and Cd‐IV (−8.617 eV) shifts toward the Fermi level compared with Cd‐I (−8.628 eV), which implies that defective Cd may exhibit enhanced catalytic performance. To obtain deeper insight into the differences among Cd‐I, Cd‐II, Cd‐III, and Cd‐IV, DFT calculations were employed to assess the intermediate variation (such as Cd‐^*^OO, Cd‐^*^OOH, and Cd‐^*^O) and energy barrier during the ORR reaction. Of particular interest, the intact Cd (Cd‐I) exhibits a higher Gibbs free energy as compared to defective Cd‐II, Cd‐III, and Cd‐IV when transitioning from ^*^OOH to H_2_O_2_ (Figure 6f), which suggested that the defective Cd‐II, Cd‐III, and Cd‐IV could offer important advantages over that intact Cd‐I for H_2_O_2_ production (Figures S24–S27, Supporting Information). In summary, our simulation results indicate that defective catalysts indeed influence electrocatalytic performance by adjusting the electronic structure of neighboring atoms and consequently the energy barriers of the 2*e* ^−^ ORR intermediates, providing theoretical support for the efficient 2*e* ^−^ ORR activity of Cd in our experimental results.

## 3 Conclusion

In summary, we have demonstrated, through a combined experimental and theoretical approach, that the catalyst of Fenton‐inactive Cd(B) enabled high electrochemical O_2_‐to‐H_2_O_2_ productivity rates without significant activity loss in long‐term catalytic reactions. Subsequently, the in situ generated H_2_O_2_ could exhibit high selectivity in oxidizing cinnamaldehyde, which was different from that of commercial products. Our findings resolve the dilemma of balancing durability and catalytic activity in conventional *d* ^10^‐electron catalysts, which not only provide important insights into the development of sustainable electrochemical systems for H_2_O_2_ production but also demonstrate the potential of the Fenton‐inactive catalysts. Further research in this area could focus on optimizing the catalyst's performance and exploring its potential applications in various industries.

## Conflict of Interest

The authors declare no conflict of interest.

## Author Contributions

Y.W., H.W., and Y.Y. contributed equally to this work.

## Supporting information



Supporting Information

## Data Availability

The data that support the findings of this study are available from the corresponding author upon reasonable request.
