# Supplementary material for: Fenton‐Inactive Cd Enables Highly Selective O2‐Derived Domino Reaction
Source: Adv Sci (Weinh). 2024 Nov 8;11(47):2407051. doi: 10.1002/advs.202407051 (PMC11653596; doi:10.1002/advs.202407051)
Supplement: Supplementary file 1 — Supporting Information [file ADVS-11-2407051-s001.pdf]

ADVANCED SCIENCE
Open Access

Supporting Information

for *Adv. Sci.*, DOI 10.1002/advs.202407051

Fenton-Inactive Cd Enables Highly Selective O_2-Derived Domino Reaction

Yitong Wang, Huilin Wang, Yulu Yang, Zhaomin Hao, Ruiping Deng, Qingsong Dong, Qingchao Liu, Hongpeng You* and Shuyan Song**

Supporting Information

Fenton-Inactive Cd Enables Highly Selective O_2-Derived Domino Reaction

Yitong Wang, Huilin Wang, Yulu Yang, Zhaomin Hao, Ruiping Deng, Qingsong Dong, Qingchao Liu , Hongpeng You*, Shuyan Song**

Content

Experimental Section....4

Synthesis of Cd(B) 4

Synthesis of Cd(B)-1, Cd(B)-2 and Cd(B)-3 4

Synthesis of Cd(C) 4

Synthesis of Cd(H) 5

Characterization....5

Electrochemical measurements 6

Theoretical section 8

Figure S1 SEM image of the Cd(B)....10

Figure S2 XRD patterns of the Cd(B). 11

Figure S3 XRD patterns of the Cd(B), Cd(B)-1, Cd(B)-2, Cd(B)-3. 12

Figure S4 SEM image of the Cd(C)....13

Figure S5 SEM image of the Cd(H). 14

Figure S6 Energy dispersive X-ray (EDX) elemental mapping images of Cd(C)....15

Figure S7 Energy dispersive X-ray (EDX) elemental mapping images of Cd(H)....16

Figure S8 The standard curve of inductively coupled plasma (ICP) measurements that based on the known concentration of B and Cd elements....17

Figure S9 Comparison of XPS patterns for B1s between Cd(B) and pure Cd. 18

Figure S10 SAED pattern of Cd(B)....19

Figure S11 Average oxidation state of Cadmium in Cd(B) from Cadmium K-edge XANES.20

Figure S12 $\kappa^3\chi(\kappa)$ oscillations of Cd K-edge EXAFS analysis for the references....21

Figure S13 The EXAFS fitting for Cd-foil, CdO and Cd(B). 22

Figure S14 Comparison of linear sweep voltammetry of RRDE measurement for Cd(C), Cd(H). 23

Figure S15 Comparison of electrochemical ORR performance of B-doped Cd with different B concentrations....24

Figure S16 The cyclic voltammogram (CV) curves and calculated electrochemical active surface area (ECSA) of Cd(B), Cd(C) and Cd(H). 25

Figure S17 The H_2O_2 concentrations and Faradaic efficiency of Cd(B), Cd(C) and Cd(H) after 1 h. 26

Figure S18 The H_2O_2 concentrations and Faradaic efficiency of Cd(B), Cd(B)-1, Cd(B)-2 and Cd(B)-3 after 1 h. 27

Figure S19 The concentrations of H_2O_2 at different operating currents after 1 h. 28

Figure S20 Schematic diagram of the solid-electrolyte for H_2O_2 production.......................... 29

Figure S21 Comparison of XPS signals for Cd3d, B1s, C1s, and O1s XPS before and after ORR. 30

Figure S22 Comparison of XRD signals before and after 2e^- ORR. 31

Figure S23 comparison of H_2O_2 concentration before and after adding the scavenger (1,4-benzoquinone, BQ) for *OO. 32

Figure S24 Diagram of calculated free energy for Cd-I... 33

Figure S25 Diagram of calculated free energy for Cd-II. 34

Figure S26 Diagram of calculated free energy for Cd-III. 35

Figure S27 Diagram of calculated free energy for Cd-IV. 36

Table S1 EXAFS fitting parameters at the Cd K-edge of various samples. (S02=0.9). 37

Table S2 Comparison of 2e^- ORR performance for Cd(B) with recently reported materials. . 38

Table S3 Comparison of the 2e^- ORR performance for Cd(B) with similar materials. 39

References 40

Experimental Section

Synthesis of Cd(B)

The Cd(B) catalyst was prepared by a wet chemical method using cadmium nitrate ($Cd(NO_3)_2·4H_2O$) and sodium borohydride ($NaBH_4$) as precursors. Firstly, dissolve 1 mmol of $Cd(NO_3)_2·4H_2O$ in 2 mL of ice water, and $Cd(NO_3)_2·4H_2O$ solution was injected rapidly into frozen $NaBH_4$ (5 M, 2 mL) solution until no bubbles formed. The centrifuged precipitate was then washed three times with distilled water and acetone to completely remove the unreacted precursors and other by-products. Finally, the powder of Cd(B) was dried under vacuum overnight at 60°C.

Synthesis of Cd(B)-1, Cd(B)-2 and Cd(B)-3

Different concentrations of $Cd(NO_3)_2$ solutions (2 mL each) were prepared: Cd(B)-1 with 400 mg $Cd(NO_3)_2$, Cd(B)-2 with 200 mg $Cd(NO_3)_2$, and Cd(B)-3 with 100 mg $Cd(NO_3)_2$. Each solution was separately added to an identical $NaBH_4$ solution (5 M, 2 mL) and allowed to react until gas evolution ceased. The resulting precipitates were collected by centrifugation and washed three times with distilled water and acetone to remove any unreacted precursors and by-products. Finally, the powders (Cd(B)-1, Cd(B)-2, and Cd(B)-3) were dried under vacuum at 60°C overnight.

Synthesis of Cd(C)

3 mmol trisodium citrate and 1 mmol $Cd(NO_3)_2·4H_2O$ were dissolved in distilled water, and then cadmium nitrate solution was injected into trisodium citrate solution until a white precipitate was formed after 30 min. The resulting powder was washed three times with distilled water and acetone to remove precursors and other

by-products, and the product was dried under vacuum overnight at 60°C. To get the final sample of Cd(C), the dried product was annealed in an Ar atmosphere at 350°C for 90 min to remove the organic molecules.

Synthesis of Cd(H)

1 mmol $Cd(NO_3)_2{\cdot}4H_2O$ was dissolved in 2 mL distilled water, and then 1 mL $N_2H_4{\cdot}H_2O$ was measured with a pipette gun and added to $Cd(NO_3)_2{\cdot}4H_2O$ solution. After 12 hours of reaction, centrifuge and wash the powder with distilled water to remove impurities. The final powder was dried under a vacuum overnight at 60°C to get the sample of Cd(H).

Characterization

The crystalline structure of the sample was measured by a Bruker D8 Focus X-ray diffractometer (XRD) with Cu Kα radiation (λ = 0.154 nm), a voltage of 40 kV and an electric current of 40 mA. The X-ray photoelectron spectra (XPS) were measured with VG ESCALAB MK (VK Company, UK) at room temperature using an Al Kα X-ray source at 12 kV and 20 mA. Energy dispersive spectroscopy (EDS) data were collected as an ensemble measurement in the FE-SEM. An energy-dispersive X-ray spectroscopy detector was used to analyze the elemental composition. A transmission electron microscope (TEM) was performed using an FEI Tecnai G2 S-Twin instrument with a field emission gun operating at 200 kV. The atomic image was filmed by a double-corrected spherical aberration electron microscope (FEI Theims Z). *In-situ* Raman spectroscopy was carried out by a Renishaw inVia Raman spectrometer equipped with a 532 nm He-Ne laser, ×50 objective, monochromator (600 grooves/mm grating) and a charge-coupled device detector. The signal

acquisition time is 90 seconds for each sample. The boron contents doped into cadmium were determined by inductively coupled plasma-optical emission spectroscopy (ICP-OES) by using a PerkinElmer Optima 2100DV ICP-OES instrument. Beamline BL14W1 specializes in X-ray absorption fine structure (XAFS). It has been open for users since May 2009. It is tunable by two types of double-crystal monochromator for covering photon energy from 4.5 keV to 50 keV. K-edge absorption of Titanium up to Lanthanum can be studied. Other heavier atomic species can be investigated via L edges. The bulk of our scientific program is in catalysis, materials science and environmental research. The ^{1}HNMR spectra were measured on a Bruker 500M NMR spectrometer. Mass spectra measurement was analyzed on a GC-MS-QP2010 S E spectrometer (Shimadzu).

Electrochemical measurements

The Princeton PARSTAT3000A-DX electrochemical workstation was used in all electrochemical experiments in a solid-electrolyte (SE) reactor. Electrochemical measurements were performed at room temperature in a standard three-electrode system. An IrO_2-coated titanium electrode and a saturated calomel electrode (SCE) served as the counter electrode and the reference electrode, respectively. The cathode side (with 1.5 cm * 1.5 cm surface area) was supplied with an oxygen/water mixture of 90 sccm of O_2 gas and 10.8 mL min^{-1} of DI water, a solution containing 1 M Na_2SO_4 flowed into the SE layer controlled by a syringe pump. The anode side was circulated with 0.5 M H_2SO_4 at 2.7 mL min^{-1}. In the middle chamber, the styrene-divinylbenzene sulfonated copolymer Dowex 50WX8 hydrogen form (Sigma-Aldrich) cation conductor was employed as the solid electrolyte.

In all measurements, the SCE reference electrode was calibrated to a reversible

hydrogen electrode (RHE):

E(RHE) = E(SCE) + 0.241 + 0.059×pH

The 2e^- ORR performance was quantified by the number of H_2O_2 formed using a rotating ring-disk electrode (RRDE) at 900 rpm. in an oxygen-saturated 0.1 M K_2SO_4 electrolyte. The RRDE (AFE7R9GCPT, Pine Research Instrumentation, USA) includes a glassy carbon rotation disk electrode (disk area: 0.196 cm^2) and a Pt ring (ring area: 0.2 cm^2). To check 2e^- ORR performance, 2.5 mg of Cd(B) electrocatalysts were dispersed in 740 μL distilled water, 240 μL ethanol, and 20 μL Nafion solution (5.0 wt%). After sonication for 30 min, 5.0 μL of the as-obtained suspension was pipetted onto the disk electrode. All the electrocatalysts were stabilized in advance by cyclic voltammetry (CV) with a scan rate of 50 mV s^{-1} and then linear sweep voltammetry (LSV) was conducted at a scan rate of 5.0 mV s^{-1}. The ring electrode was set at a constant potential of 1.20 V vs. RHE according to the published paper,[5, 48] which could avoid ORR currents at the ring, allowing only H_2O_2 oxidation. The selectivity (%) and the electron transfer number (N) of H_2O_2 were calculated using the following equations:

$$H_2O_2\ (\%) = 200 \times \frac{I_r/N}{I_d + I_r/N}$$

$$n = 4 \times \frac{I_d}{I_d + I_r/N}$$

where I_r is the ring current, I_d is the disk current, and N is the current collection efficiency of the Pt ring electrode (The collection efficiency of the RRDE electrode used was determined by using the reversible $[Fe(CN)_6]^{4-}/[Fe(CN)_6]^{3-}$ system). The H_2O_2-proof reaction was as follows:

$2Ce^{4+} + H_2O_2 \rightarrow 2Ce^{3+} + 2H^+ + O_2 \uparrow$

$2MnO^{4-} + 5H_2O_2 + 6H^+ \rightarrow 2Mn^{2+} + 5O_2 \uparrow + 8H_2O$

Theoretical section

DFT calculations in this study were carried out using the Vienna Ab Initio Simulation Package (VASP) code. The ion-electron interaction was described with the projector-augmented wave (PAW) method. The electron exchange-correlation was modeled using the Perdew, Burke, and Ernzerhof (PBE) functional within the framework of the generalized gradient approximation (GGA). Electron localization function (ELF) is one of the important ways to study the electronic structure, which can effectively characterize the degree of electron localization in catalysts. To get the ELF, we first obtained the ELFCAR file through VASP calculation and then used the VASTA to obtain the schematic diagram. For the differential charge density, we followed a technique similar to that of ground state energy calculation: $\Delta\rho = \rho AB - \rho A - \rho B$. Where, ρAB, ρA and ρB are the charge densities of Cd−*O, Cd and *O, respectively. To get appropriate Δρ, the NGX, NGY, NGZ, NGXF, NGYF and NGZF of ρAB, ρA and ρB must be consistent with each other in the same catalyst. To estimate the adsorption energies of different intermediates, we calculated the reaction energies of each intermediate and corrected them for zero-point energy (ZPE) and entropy (TS) using the equation: $\Delta G = \Delta E + \Delta ZPE - T\Delta S$. To gain atomic-level insight into the effect of catalysts-to-ORR correlation, four atomic models (Cd-I, Cd-II, Cd-III and Cd-IV) were studied by referring to the coordination environment of Cd. A vacuum region of about 20 Å was used to decouple the periodic replicas. The energy cutoff for the plane-wave basis set was 400 eV and a (2×2×1) k-point sampling was used following the Monkhorst-Pack scheme. In all calculations, the convergence

criterion of the electronic structures was set to 10^{-5} eV, and the atomic positions were allowed to relax until the forces were less than 0.01 eV $Å^{-1}$.

Figure S1 SEM image of the Cd(B).

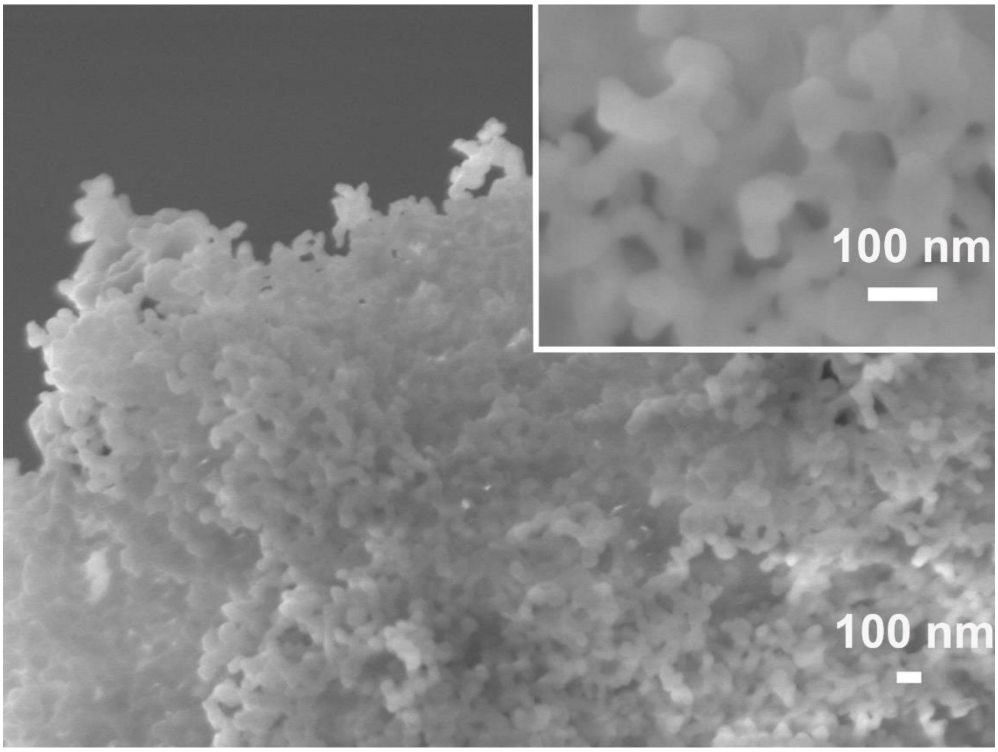

Figure S2 XRD patterns of the Cd(B).

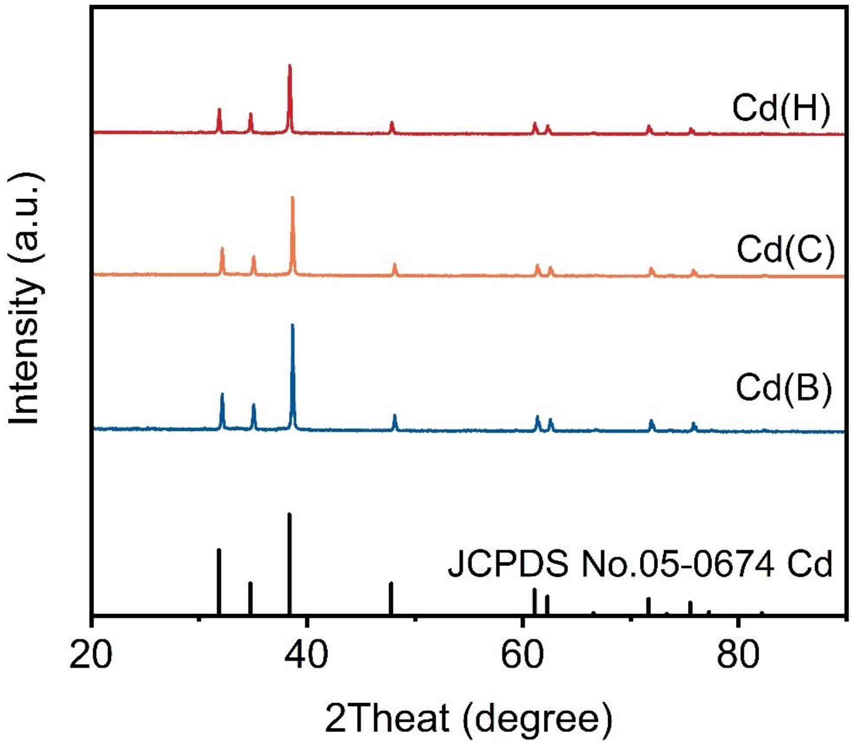

Figure S3 XRD patterns of the Cd(B), Cd(B)-1, Cd(B)-2, Cd(B)-3.

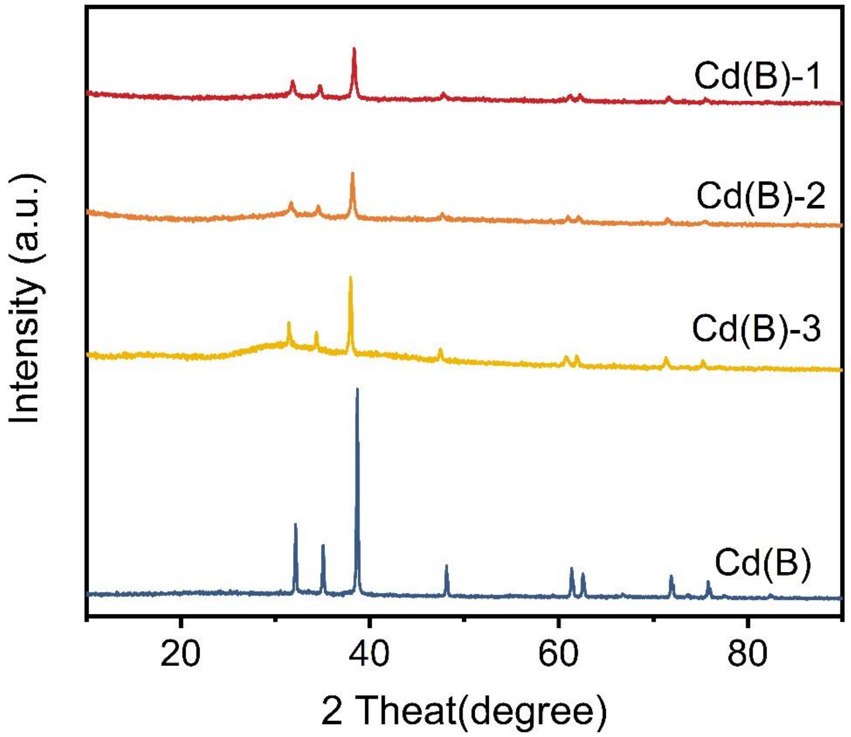

Figure S4 SEM image of the Cd(C).

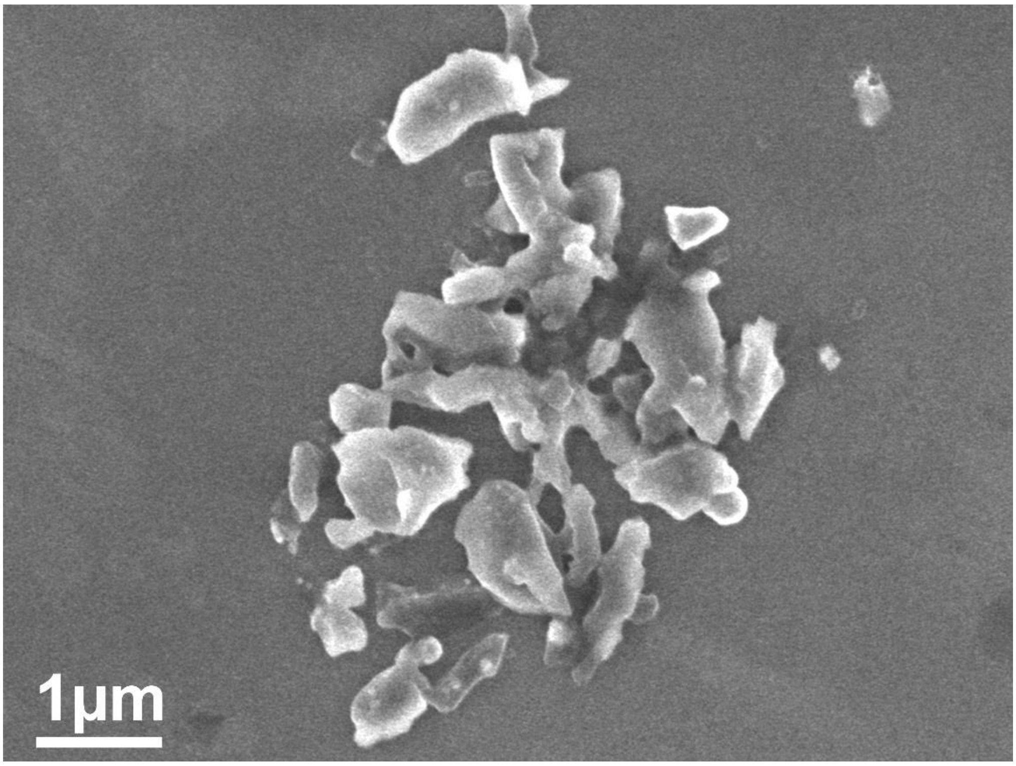

Figure S5 SEM image of the Cd(H).

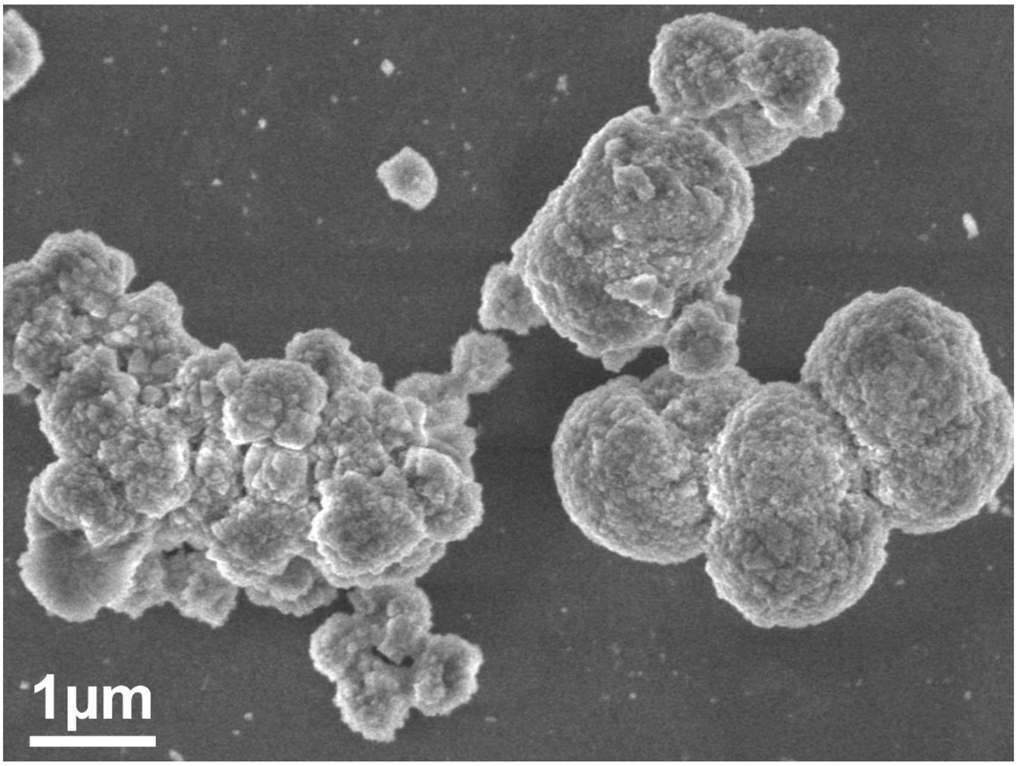

Figure S6 Energy dispersive X-ray (EDX) elemental mapping images of Cd(C).

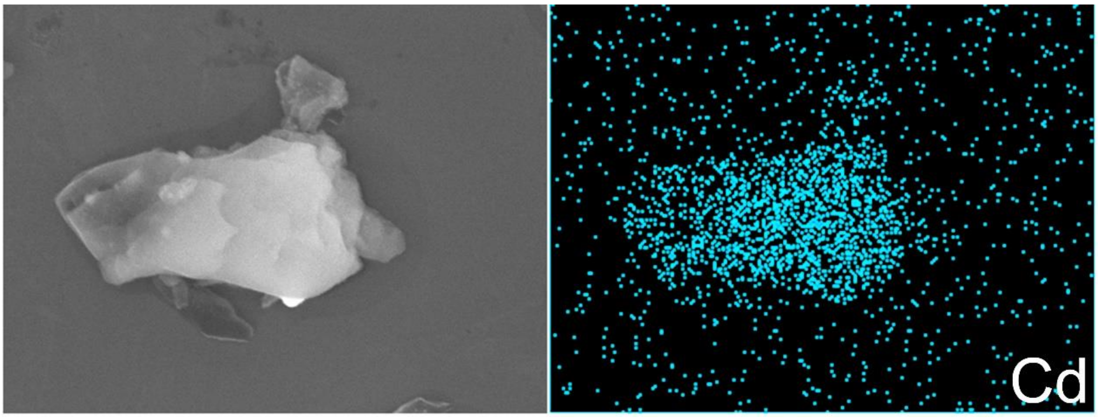

Figure S7 Energy dispersive X-ray (EDX) elemental mapping images of Cd(H).

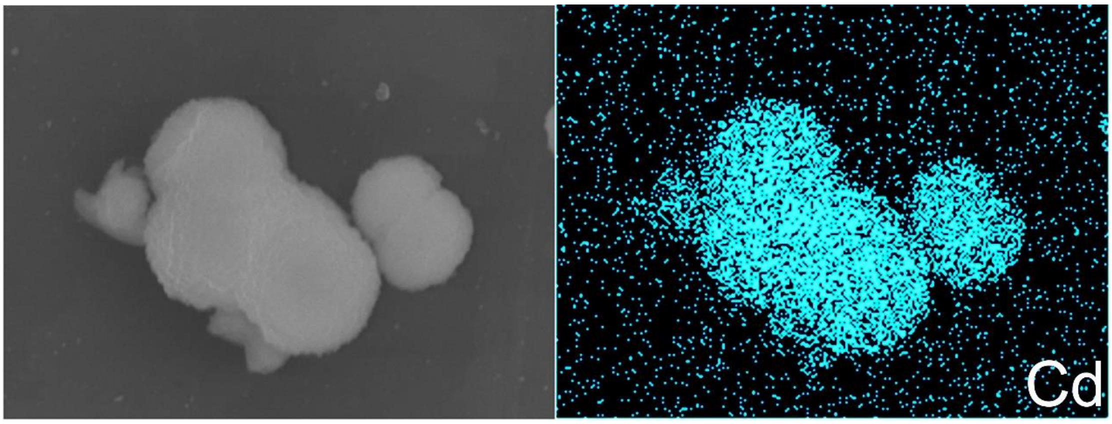

Figure S8 The standard curve of inductively coupled plasma (ICP) measurements that based on the known concentration of B and Cd elements.

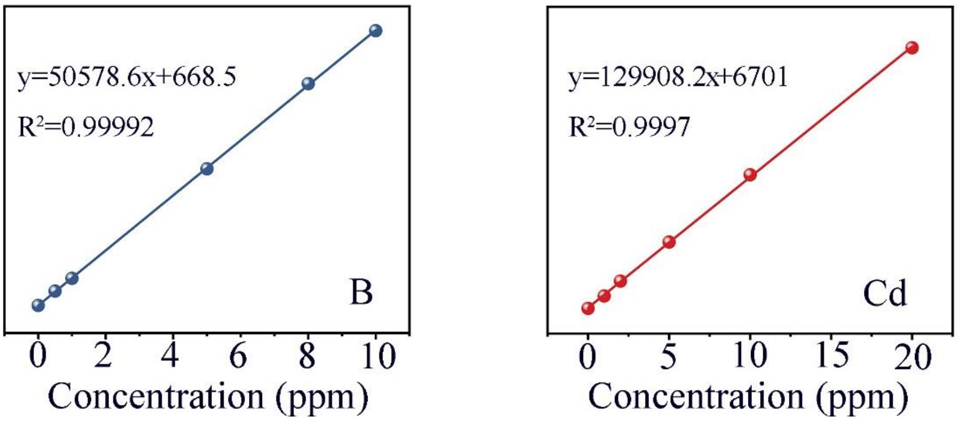

Figure S9 Comparison of XPS patterns for B1s between Cd(B) and pure Cd.

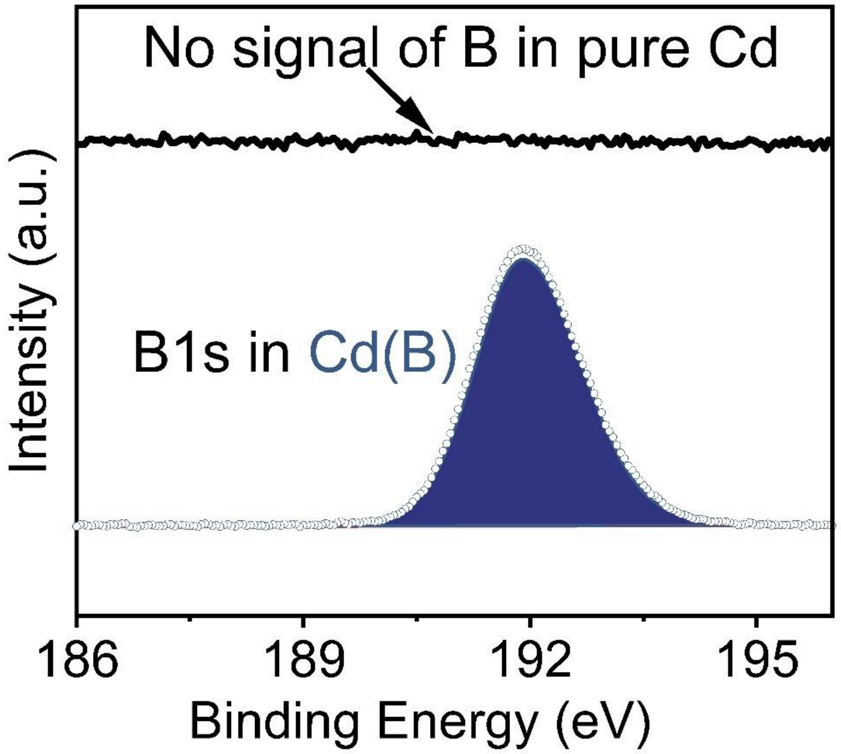

Figure S10 SAED pattern of Cd(B).

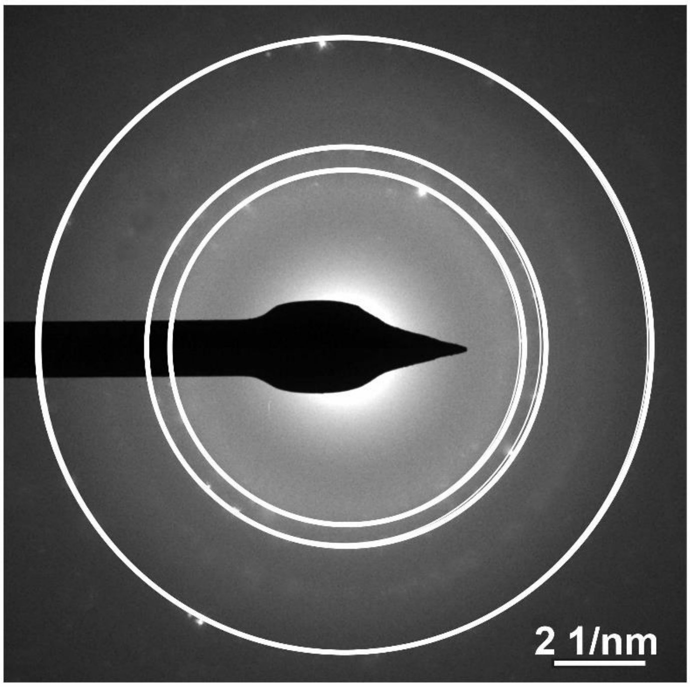

Figure S11 Average oxidation state of Cadmium in Cd(B) from Cadmium K-edge XANES.

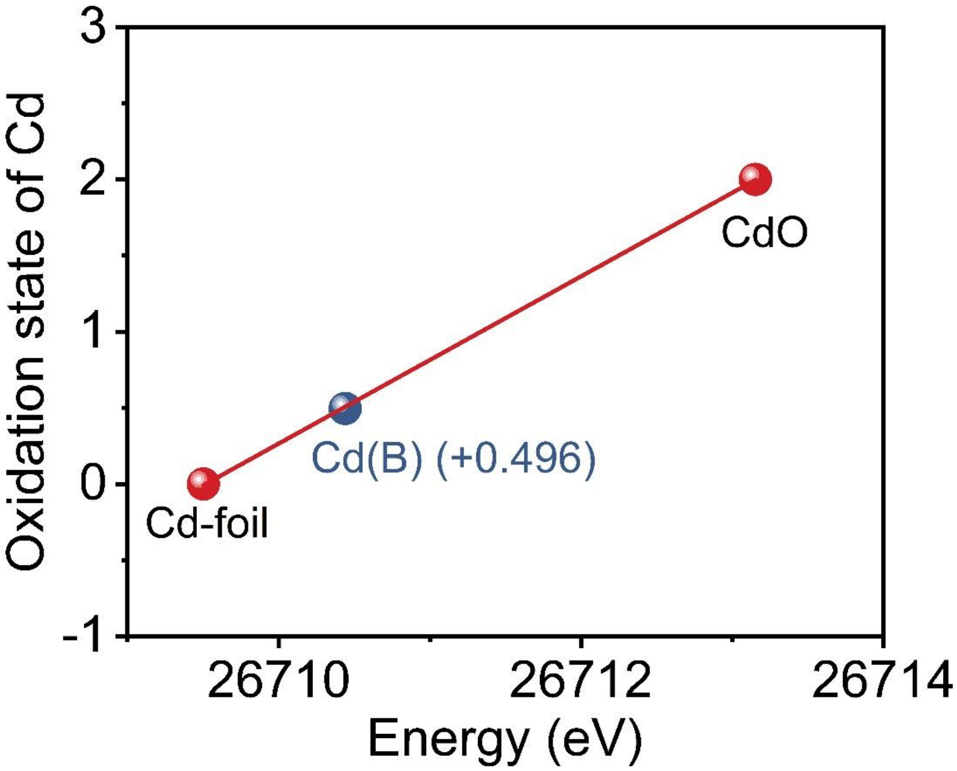

Figure S12 $\kappa^3\chi(\kappa)$ oscillations of Cd K-edge EXAFS analysis for the references.

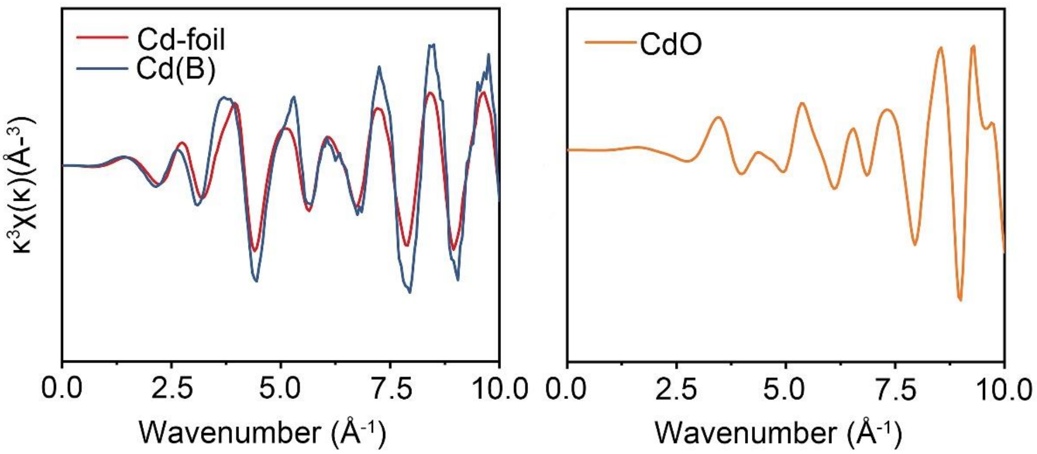

Figure S13 The EXAFS fitting for Cd-foil, CdO and Cd(B).

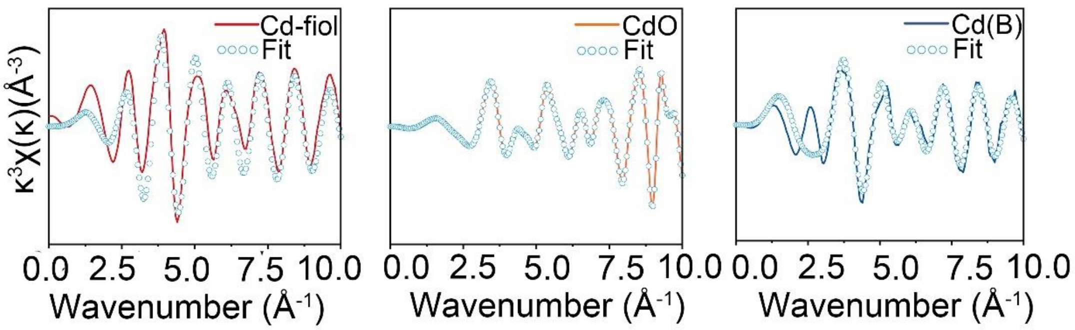

Figure S14 Comparison of linear sweep voltammetry of RRDE measurement for Cd(C), Cd(H).

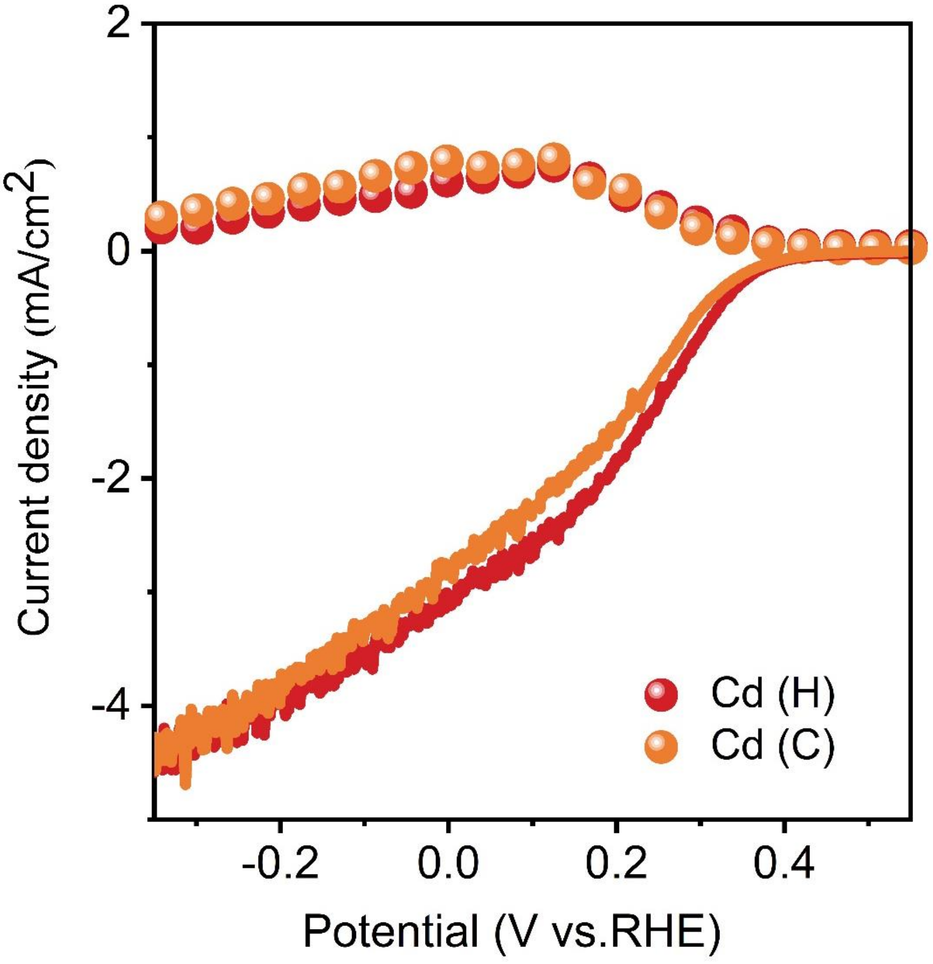

Figure S15 Comparison of electrochemical ORR performance of B-doped Cd with different B concentrations.

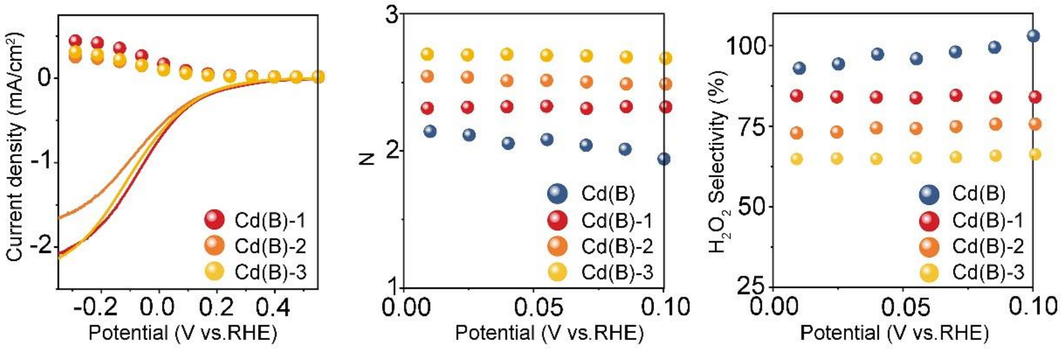

Figure S16 The cyclic voltammogram (CV) curves and calculated electrochemical active surface area (ECSA) of Cd(B), Cd(C) and Cd(H).

To get ECSA, current density (taken at the potential of 0.24 V vs. RHE, modified electrodes in the double layer region) as a function of scan rate (5, 15, 20, 30 and 50 mV s^{-1}) derived from cyclic voltammogram (CV), respectively.

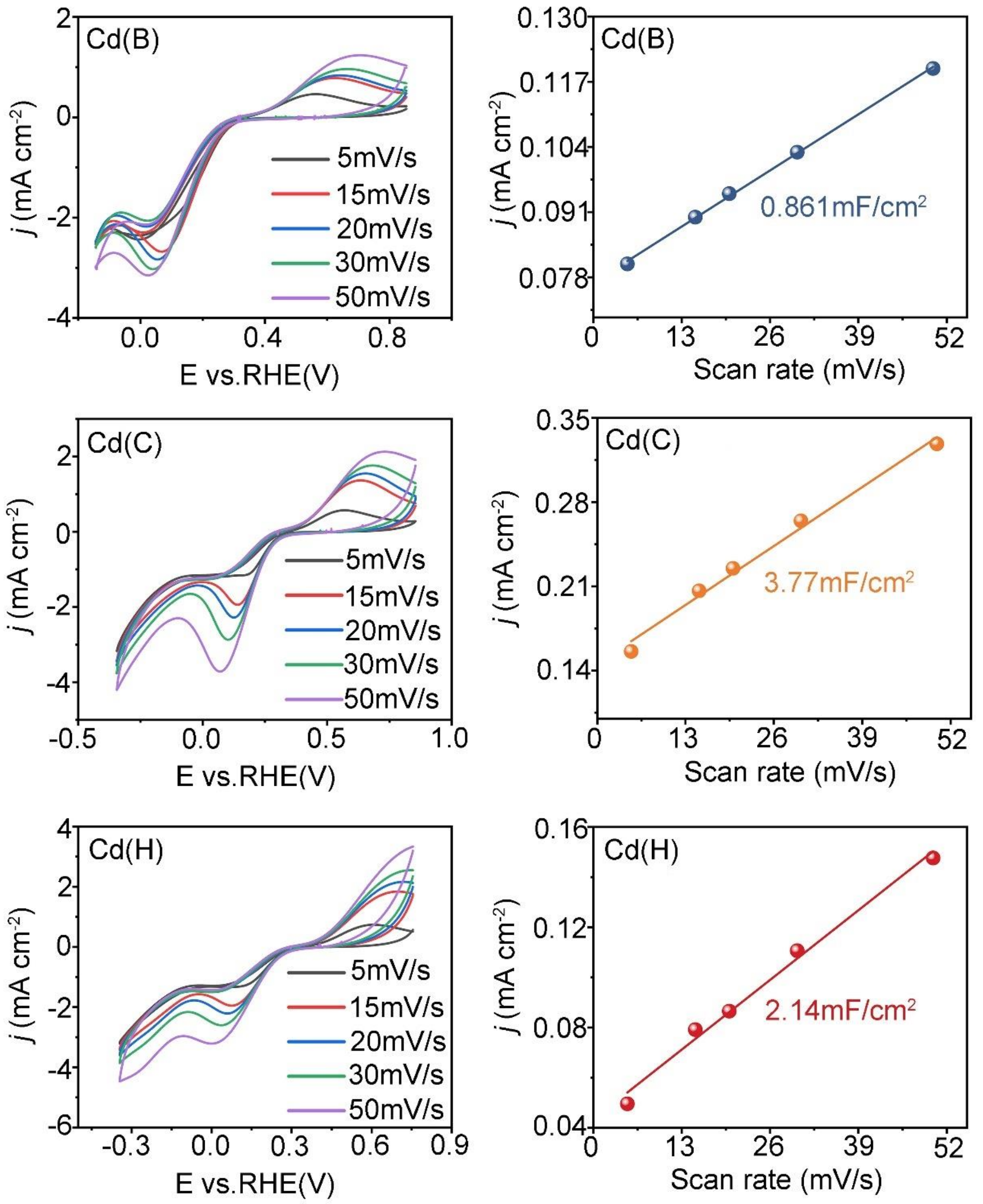

Figure S17 The H_2O_2 concentrations and Faradaic efficiency of Cd(B), Cd(C) and Cd(H) after 1 h.

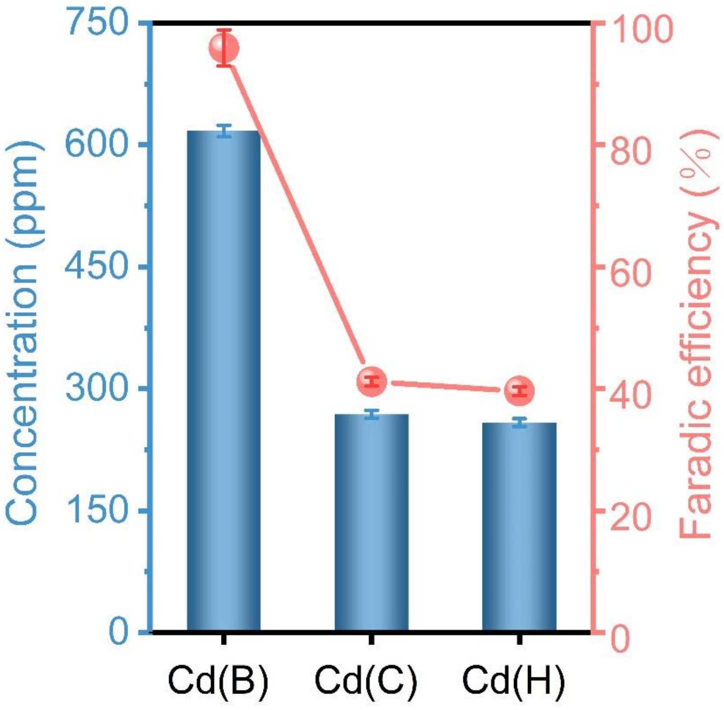

Figure S18 The H_2O_2 concentrations and Faradaic efficiency of Cd(B), Cd(B)-1, Cd(B)-2 and Cd(B)-3 after 1 h.

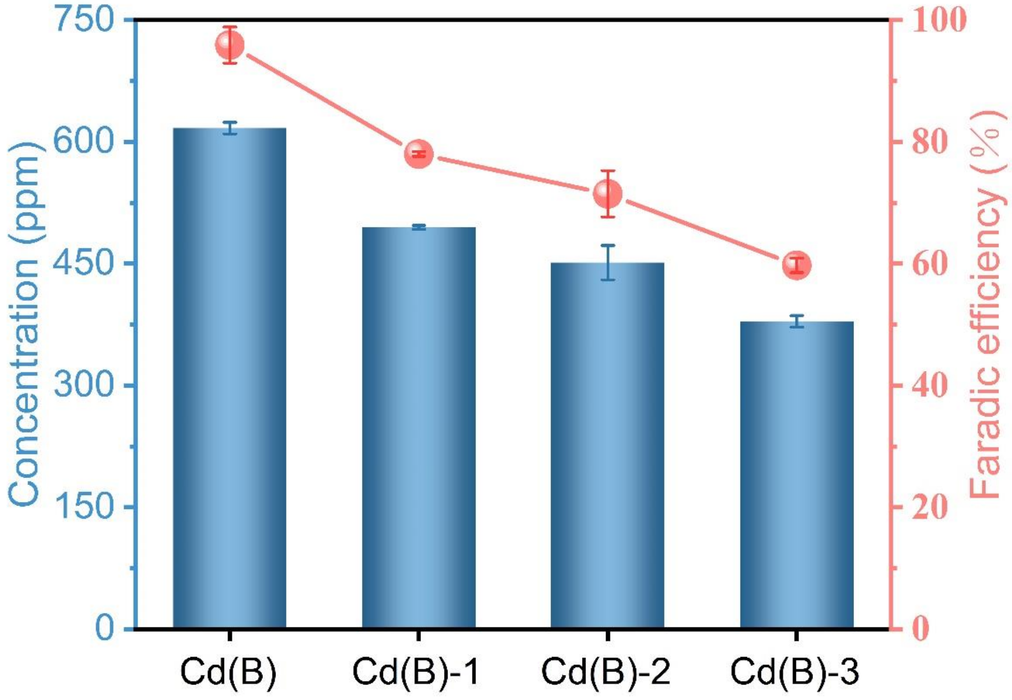

Figure S19 The concentrations of H_2O_2 at different operating currents after 1 h.

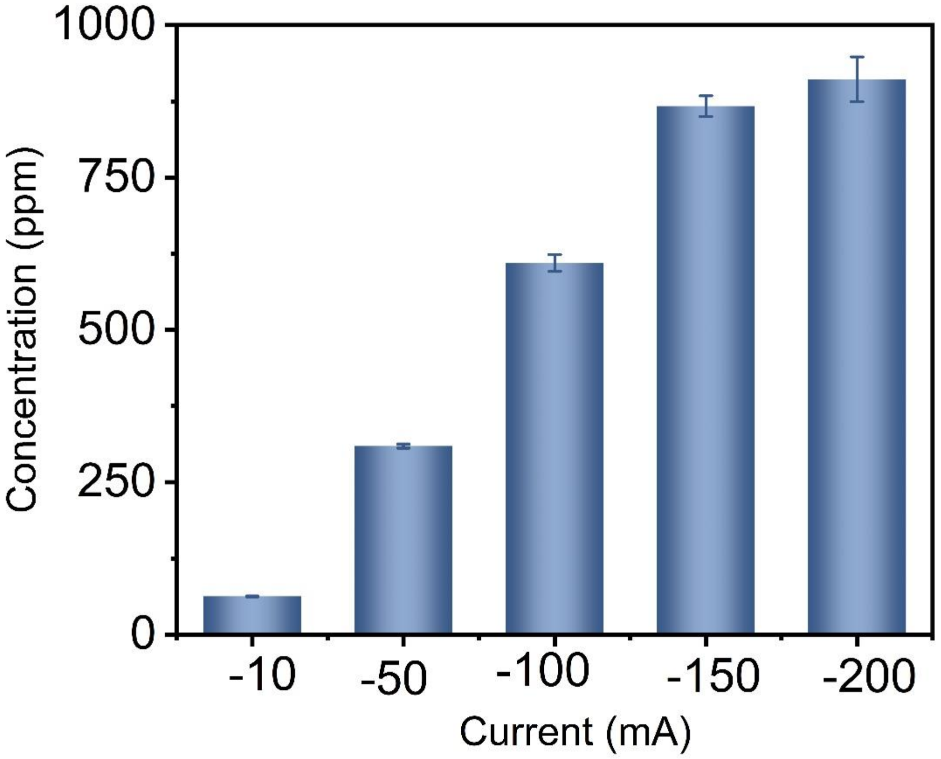

Figure S20 Schematic diagram of the solid-electrolyte for H_2O_2 production.

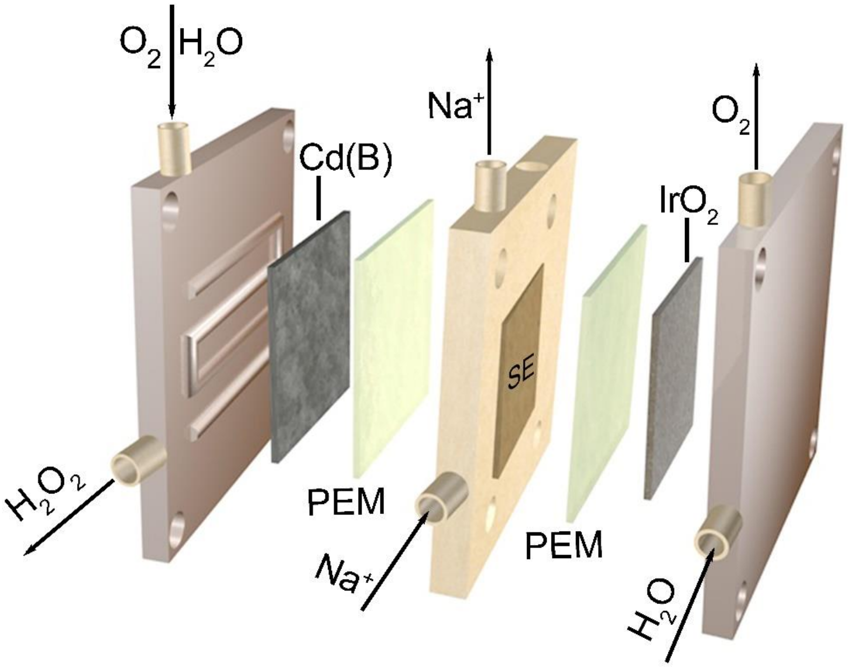

Figure S21 Comparison of XPS signals for Cd3d, B1s, C1s, and O1s XPS before and after ORR.

The XPS results showed that Cd and B were relatively stable. The appearance of the O1s peak was caused by oxygen in the air during the detection process before and after the reaction. For the C1s in XPS, the peak at 291.9 eV had a significant change in intensity mainly because it was caused by C-F in the Nafion solution before and after the reaction. The Nafion solution could be gradually washed off from the catalyst, so there was no C-F peak in the XPS after the reaction. Moreover, no C-O peak was observed in the C spectrum after the reaction, indicating that only the Cd(B) catalyst played a catalytic role in the reaction.

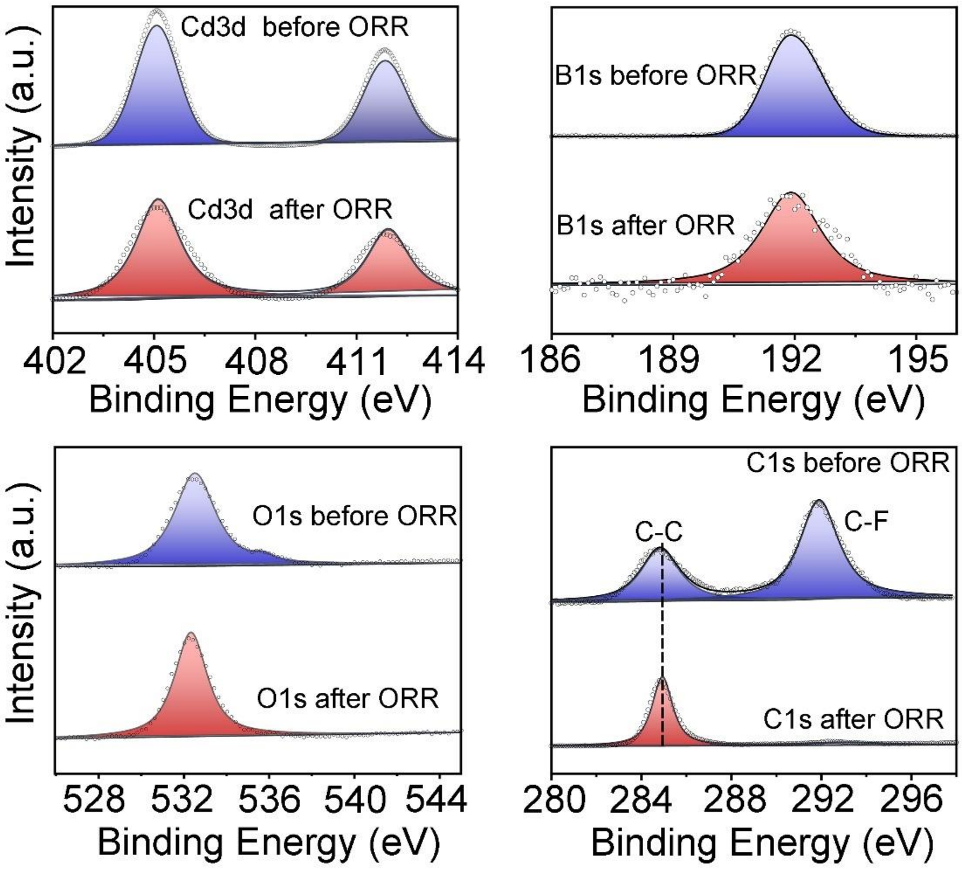

Figure S22 Comparison of XRD signals before and after 2e^- ORR.

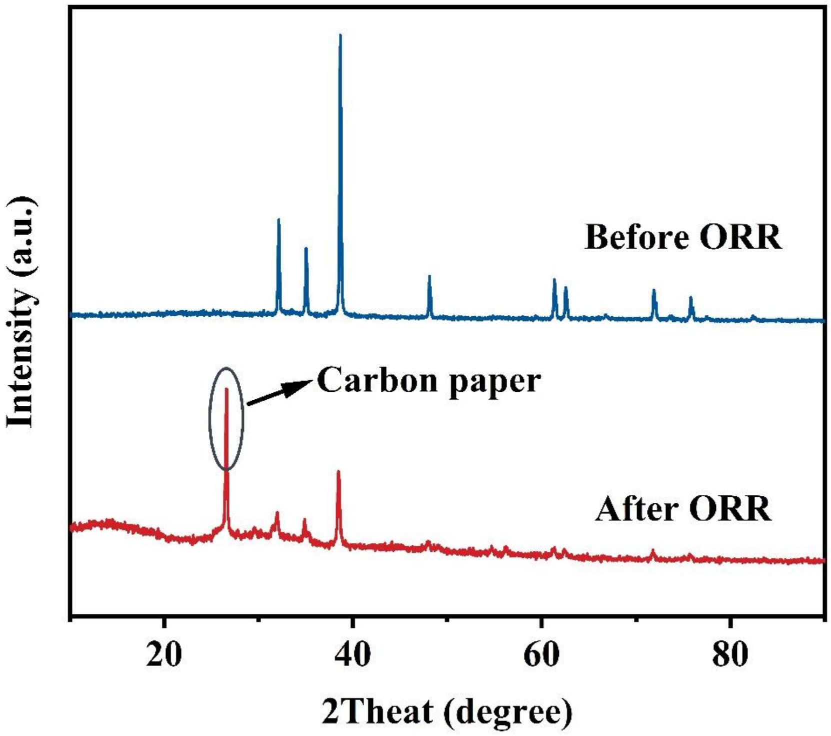

Figure S23 comparison of H_2O_2 concentration before and after adding the scavenger (1,4-benzoquinone, BQ) for *OO.

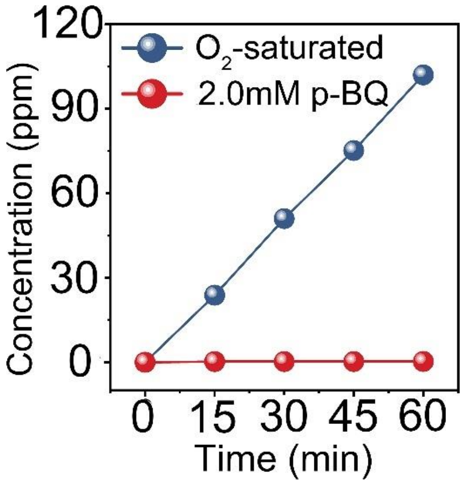

Figure S24 Diagram of calculated free energy for Cd-I.

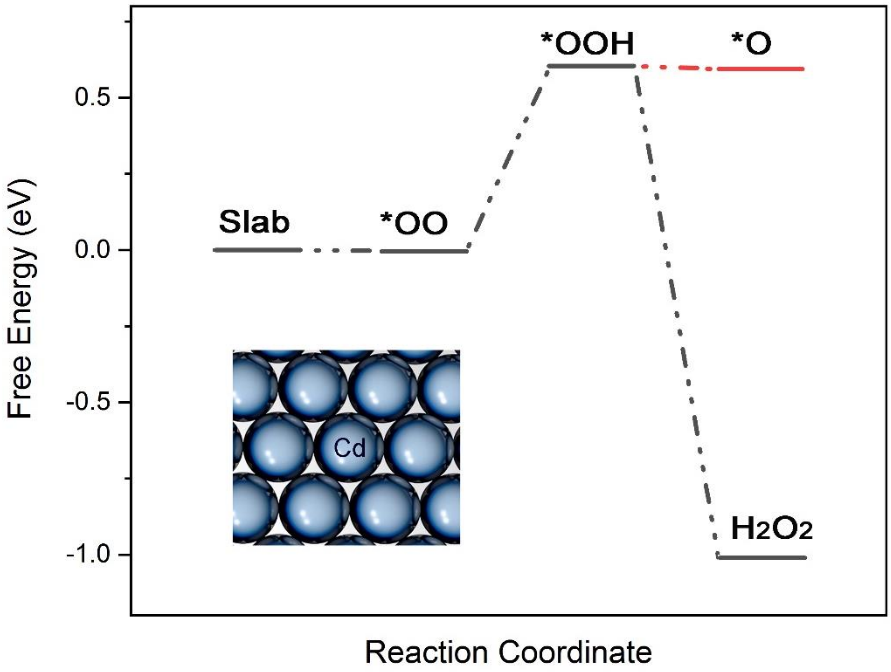

Figure S25 Diagram of calculated free energy for Cd-II.

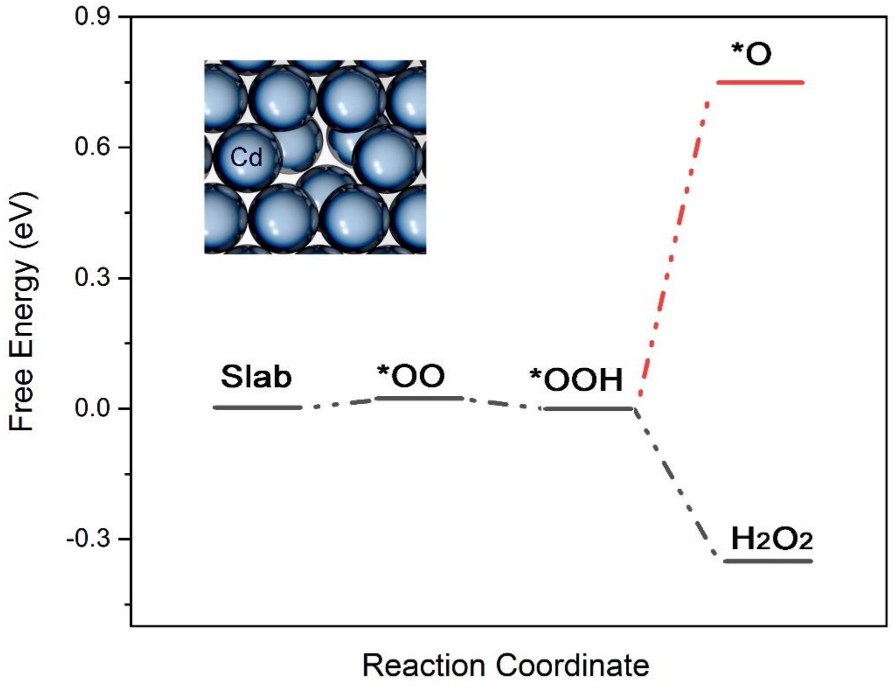

Figure S26 Diagram of calculated free energy for Cd-III.

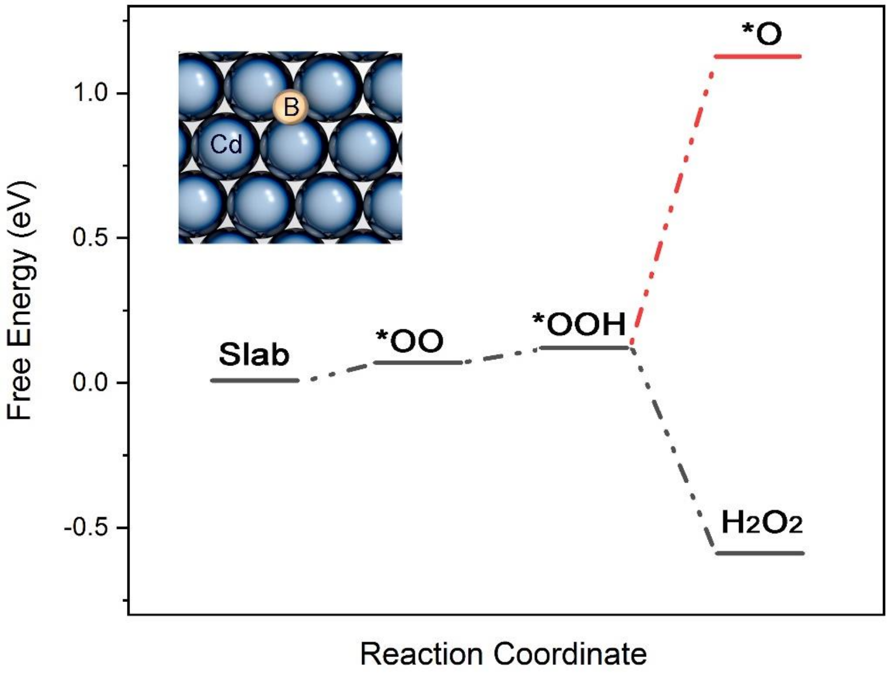

Figure S27 Diagram of calculated free energy for Cd-IV.

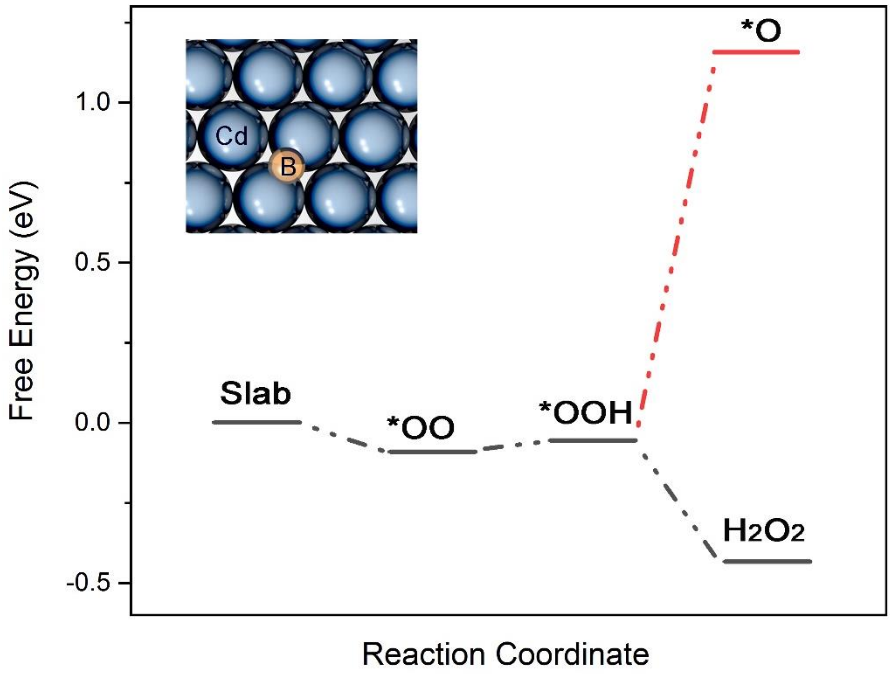

Table S1 EXAFS fitting parameters at the Cd K-edge of various samples. (S02=0.9).

Samples	Path	CN	σ^2(Å^2)	ΔE(eV)	R-factor
Cd-foil	Cd-Cd	6	0.012	-3.035	0.0078
CdO	Cd-Cd	12	0.002	0.3	0.006
	Cd-O	6	0.003		
Cd(B)	Cd-Cd	5.026	0.01	-3.0006	0.0088
	Cd-O	4.79	0.0049		

Table S2 Comparison of 2e^- ORR performance for Cd(B) with recently reported materials.

catalysts	Electrolyte	Stability	rate ($mol_{H_2O_2}g^{-1}h^{-1}$)	Applied potentials	Reference
Cd(B)	1M Na_2SO_4	100h	15.5	-0.35 V vs. RHE	**This work**
CoIn-N-C	0.1M $HClO_4$	5h	9.68	-	*Nat. Commun.* **14**, 4766 (2023)[1]
Co-SCD-2	0.1M KOH	50h	5.58	0.6V vs. RHE	*Angew.Chem.Int.Ed.* **62**, e202307355 (2023)[2]
ZnO@ZnO_2	0.1M K_2SO_4	40h	5.47	0.1V vs. RHE	*Energy Environ. Sci.* **16**, 3526 (2023)[3]
CB@Co-N-C	0.5M H_2SO_4	200h	5.04	0V vs. RHE	*Adv. Funct. Mater.* **33**, 1 (2023)[4]
CoPc-OCNT	0.1M KOH	30h	11.527	300 mA cm^{-2}	*Nat. Commun.* **14**, 172 (2023)[5]
Sb-NSCF	0.1M KOH	75h	7.46	0.55V vs. RHE	*Nat. Commun.* **14**, 368 (2023)[6]
PFC-72-Co	0.5M H_2SO_4	30h	-	0.55V vs. RHE	*Nat. Commun.* **13**, 2721 (2022)[7]
O–C(Al)	0.5MNa_2SO_4	10h	0.51	0.47V vs. RHE	*Nat. Commun.* **11**, 5478 (2020)[8]
Al_2O_3/PtP_2-600	0.1M $HClO_4$	120h	0.706	-	*Nat. Commun.* **11**, 3928 (2020)[9]
ZnCo-ZIF-C3	0.1M PBS	100h	4.35	-	*J. Am. Chem. Soc.* **145**, 7791 (2023)[10]
Co−N SAC_{Dp}	0.1M $HClO_4$	90h	0.78	0.25V vs. RHE	*J. Am. Chem. Soc.* **144**, 14505 (2022)[11]
FeSA-NS/C-700	0.1M KOH	30h	4.95	-0.5V vs. RHE	*Angew.Chem.Int.Ed.* **62**, e202306491 (2023)[12]
h-SnO_2	0.1M Na_2SO_4	20h	3.88	0V vs. RHE	*Angew.Chem.Int.Ed.* **62**, e202218924 (2023)[13]
Ni-N_2O_2/C	0.1M KOH	8h	5.9	-	*Angew. Chem. In.t Ed.* **59**, 13057 (2020)[14]
CoPc-CNT(O)	1M Na_2SO_4	100h	-	0.28V vs. RHE	*Nat.Catal.***6**, 234 (2023) [15]
a-$PdSe_2$ NPs	0.1M KOH	25h	3.24	0.2V vs. RHE	*Adv. Mater.* **35**, e2208101 (2023)[16]
NiNx/$CAQNH_2$	0.1M KOH	72h	-	0.5V vs. RHE	*Adv. Mater.* **34**, e2104891 (2022)[17]
CoPorF/CNT	0.1M $HClO_4$	48h	10.76	-	*Energy Environ. Sci.* **16**, 446 (2023)[18]

Table S3 Comparison of the 2e^- ORR performance for Cd(B) with similar materials.

catalysts	Electrolyte	Stability	rate ($mol_{H_2O_2} g^{-1} h^{-1}$)	Applied potentials	Reference
Cd(B)	1M Na2SO4	100h	15.5	-0.35V vs. RHE	This work
Au-Pd2Hg5	0.1M HClO4	1h	0.00149	0.25V vs. RHE	*Adv. Mater. 35, 2211512 (2023)*
NC-Ag/NHCS	0.1M $HClO_4$	48h	0.408	0.7V vs. RHE	*Nano Res.* **15**, 5842 (2022)
NiB_2	0.1M KOH	12h	4.753	0.4V vs. RHE	*Adv. Mater.* **34**, 2202995 (2022)
Ni/MOF NSs	0.1M KOH	11h	6.5	0.6V vs. RHE	*Angew. Chem. Int. Ed.* **59**, 14373 (2020)
CoPc-OCNT	0.1M KOH	30h	11.527	-	*Nat. Commun.* **14**, 172 (2023)
Sb-NSCF	0.1M KOH	75h	7.46	0.55V vs. RHE	*Nat. Commun.* **14**, 368 (2023)

References

[1] J. Du, G. Han, W. Zhang, L. Li, Y. Yan, Y. Shi, X. Zhang, L. Geng, Z. Wang, Y. Xiong, G. Yin, C. Du, *Nat. Commun.* **2023**, *14*, 4766.

[2] D. Qi, J. Xu, Y. Zhou, H. Zhang, J. Shi, K. He, Y. Yuan, J. Luo, S. Wang, Y. Wang, *Angew Chem. Int. Ed.* **2023**, *62*, e202307355.

[3] Y. Zhou, L. Xu, J. Wu, W. Zhu, T. He, H. Yang, H. Huang, T. Cheng, Y. Liu, Z. Kang, *Energy Environ. Sci.* **2023**, *16*, 3526.

[4] Y. X. Du, Q. Yang, W. T. Lu, Q. Y. Guan, F. F. Cao, G. Zhang, *Adv. Funct. Mater.* **2023**, *33*, 2300895

[5] P. Cao, X. Quan, X. Nie, K. Zhao, Y. Liu, S. Chen, H. Yu, J. G. Chen, *Nat. Commun.* **2023**, *14*, 172.

[6] L. Fan, Y. Zhao, L. Chen, J. Chen, J. Chen, H. Yang, Y. Xiao, T. Zhang, J. Chen, L. Wang, *Nat. Catal.* **2023**, *6*, 585.

[7] X. Zhao, Q. Yin, X. Mao, C. Cheng, L. Zhang, L. Wang, T. F. Liu, Y. Li, Y. Li, *Nat. Commun.* **2022**, *13*, 2721.

[8] Q. Yang, W. Xu, S. Gong, G. Zheng, Z. Tian, Y. Wen, L. Peng, L. Zhang, Z. Lu, L. Chen, *Nat. Commun.* **2020**, *11*, 5478.

[9] H. Li, P. Wen, D. S. Itanze, Z. D. Hood, S. Adhikari, C. Lu, X. Ma, C. Dun, L. Jiang, D. L. Carroll, Y. Qiu, S. M. Geyer, *Nat. Commun.* **2020**, *11*, 3928.

[10] C. Zhang, L. Yuan, C. Liu, Z. Li, Y. Zou, X. Zhang, Y. Zhang, Z. Zhang, G. Wei, C. Yu, *J. Am. Chem. Soc.* **2023**, *145*, 7791.

[11] S. Chen, T. Luo, X. Li, K. Chen, J. Fu, K. Liu, C. Cai, Q. Wang, H. Li, Y. Chen, C. Ma, L. Zhu, Y. R. Lu, T. S. Chan, M. Zhu, E. Cortes, M. Liu, *J. Am. Chem. Soc.* **2022**, *144*, 14505.

[12] Y. Li, J. Chen, Y. Ji, Z. Zhao, W. Cui, X. Sang, Y. Cheng, B. Yang, Z. Li, Q. Zhang, L. Lei, Z. Wen, L. Dai, Y. Hou, *Angew Chem. Int. Ed.* **2023**, *62*, e202306491.

[13] Y. Zhang, M. Wang, W. Zhu, M. Fang, M. Ma, F. Liao, H. Yang, T. Cheng, C. W. Pao, Y. C. Chang, Z. Hu, Q. Shao, M. Shao, Z. Kang, *Angew Chem. Int. Ed.* **2023**, *62*, e202218924.

[14] Y. Wang, R. Shi, L. Shang, G. I. N. Waterhouse, J. Zhao, Q. Zhang, L. Gu, T. Zhang, *Angew Chem. Int. Ed.* **2020**, *59*, 13057.

[15] B.-H. Lee, H. Shin, A. S. Rasouli, H. Choubisa, P. Ou, R. Dorakhan, I. Grigioni, G. Lee, E. Shirzadi, R. K. Miao, J. Wicks, S. Park, H. S. Lee, J. Zhang, Y. Chen, Z. Chen, D. Sinton, T. Hyeon, Y.-E. Sung, E. H. Sargent, *Nat. Catal.* **2023**, *6*, 234.

[16] Z. Yu, S. Lv, Q. Yao, N. Fang, Y. Xu, Q. Shao, C. W. Pao, J. F. Lee, G. Li, L. M. Yang, X. Huang, *Adv. Mater.* **2023**, *35*, e2208101.

[17] X. Li, S. Tang, S. Dou, H. J. Fan, T. S. Choksi, X. Wang, *Adv. Mater.* **2022**, *34*, e2104891.

[18] C. Liu, Z. Yu, F. She, J. Chen, F. Liu, J. Qu, J. M. Cairney, C. Wu, K. Liu, W. Yang, H. Zheng, Y. Chen, H. Li, L. Wei, *Energy Environ. Sci.* **2023**, *16*, 446
